# Histone H1 Variants in Arabidopsis Are Subject to Numerous Post-Translational Modifications, Both Conserved and Previously Unknown in Histones, Suggesting Complex Functions of H1 in Plants

**DOI:** 10.1371/journal.pone.0147908

**Published:** 2016-01-28

**Authors:** Maciej Kotliński, Kinga Rutowicz, Łukasz Kniżewski, Antoni Palusiński, Jacek Olędzki, Anna Fogtman, Tymon Rubel, Marta Koblowska, Michał Dadlez, Krzysztof Ginalski, Andrzej Jerzmanowski

**Affiliations:** 1 Laboratory of Systems Biology, Faculty of Biology, University of Warsaw, Warsaw, Poland; 2 Institute of Biochemistry and Biophysics, Polish Academy of Sciences, Warsaw, Poland; 3 Laboratory of Bioinformatics and Systems Biology, Centre of New Technologies, University of Warsaw, Warsaw, Poland; 4 Institute of Radioelectronic and Multimedia Technology, Warsaw University of Technology, Warsaw, Poland; 5 Institute of Genetics and Biotechnology, Faculty of Biology, University of Warsaw, Warsaw, Poland; Universidad Miguel Hernández de Elche, SPAIN

## Abstract

Linker histones (H1s) are conserved and ubiquitous structural components of eukaryotic chromatin. Multiple non-allelic variants of H1, which differ in their DNA/nucleosome binding properties, co-exist in animal and plant cells and have been implicated in the control of genetic programs during development and differentiation. Studies in mammals and Drosophila have revealed diverse post-translational modifications of H1s, most of which are of unknown function. So far, it is not known how this pattern compares with that of H1s from other major lineages of multicellular Eukaryotes. Here, we show that the two main H1variants of a model flowering plant *Arabidopsis thaliana* are subject to a rich and diverse array of post-translational modifications. The distribution of these modifications in the H1 molecule, especially in its globular domain (GH1), resembles that occurring in mammalian H1s, suggesting that their functional significance is likely to be conserved. While the majority of modifications detected in Arabidopsis H1s, including phosphorylation, acetylation, mono- and dimethylation, formylation, crotonylation and propionylation, have also been reported in H1s of other species, some others have not been previously identified in histones.

## Introduction

Linker (H1) histones are abundant structural components of eukaryotic chromatin. Due to their ability to organize inter-nucleosomal linker DNA, they play a fundamental role in the stabilization of compact higher-order chromatin structure, and are thus major agents limiting the accessibility of DNA to *trans*-acting factors. They are also the most variable of the histones, with multiple non-allelic sequence variants often coexisting in the same cell. The H1 molecule consists of an evolutionarily conserved globular domain (GH1), flanked by much more variable unstructured N- and C-terminal domains, the latter being particularly rich in positively charged (Lys, Arg) residues. It is generally agreed that H1 is positioned on the outside of the nucleosome with GH1 binding in a structure-specific way near its dyad region, *via* at least two binding sites [1–3]. This enables bridging by H1 of different DNA strands, and is critical for its role in stabilization of the nucleosome. The two traditionally defined binding sites, termed site I and site II, are located on the surface of the GH1 domain and contain most of the DNA-binding residues, including several positively charged arginines and lysines [4]. Recently, an additional binding site, termed site III, was proposed, comprised mainly of the nonpolar “wing” region responsible for the recognition of methyl groups in the major groove of AT-rich DNA segments [5].

How GH1 binds to the nucleosome has not been unequivocally determined. Among the conflicting structural models, two major classes, symmetric and asymmetric, can be distinguished, which differ in the proposed location of GH1 in the nucleosome. For example, a recent symmetrical model based on high resolution mapping of H1-reconstituted mouse nucleosome interactions proposes that GH1 interacts with the DNA minor grove at the center of the nucleosome and contacts 10-bp DNA regions localized symmetrically with respect to the nucleosomal dyad. Such a location enables GH1 to interact and organize about one helical turn of DNA in each linker region of the nucleosome [1]. The same study showed that only a 7-amino acid fragment of the C-terminal domain, directly adjacent to GH1, was sufficient to generate a stem-like structure in the linker DNA [1]. On the other hand, according to another recent study using solution NMR (nuclear magnetic resonance) spectroscopy, GH1 bridges the reconstituted Drosophila nucleosome core and one 10-bp linker DNA asymmetrically, with its α3 helix facing the nucleosomal DNA near the dyad axis. In addition, two short regions in the C-terminal domain of H1 and the C-terminal domain of one of the two H2A core histones were also shown to be involved in stabilization of the H1-nucleosome complex [2]. The asymmetric binding of histone H1 to the nucleosome and its role in the formation of a more compact structure was also postulated in a recent report that examined higher order chromatin fibers reconstituted using *X*. *leavis* histones in the presence of H1 by 11-Å-resolution cryogenic electron microscopy (cryo-EM) [3].

H1 binds chromatin in a complex and dynamic way. As recently demonstrated by FRAP (Fluorescence Recovery after Photobleaching) analysis in living cells, strong cooperativity between GH1 and the C-terminal domain, with one facilitating the binding of the other, is critical for efficient binding of H1 to chromatin [6]. The N-terminal domain is generally considered to be not important for chromatin binding [7]. Despite H1’s role as a crucial negative regulator restricting the accessibility of chromatin DNA to various proteins [8], it has also been shown to be involved in the recruitment, mostly *via* interactions with distant parts of the C- and N-terminal domains, of at least 20 different proteins [7,9]. This finding suggests a possible parallel function of H1 as a mediator facilitating the access of important modifying factors to chromatin. FRAP analysis also revealed that H1 is highly mobile *in vivo*. While in general chromatin is continuously associated with H1, individual nucleosomes may be bound by or free of H1 at any given time, depending on the stoichiometry of H1 and its competitors (including other non-allelic variants of H1), and on proteins that modify H1’s binding properties [10].

There is increasing evidence that H1 histones, similarly to core histones, participate in determining the pattern of chromatin epigenetic modifications. In mouse embryonic cells, a significant reduction of H1 led to the loss of DNA methylation on regulatory sequences of several imprinted genes [11]. Interestingly, it was shown that some murine H1 variants interact with DNA methyltransferase 1 (DNMT1) and DNA methyltransferase 3B (DNMT3B) and play key role in regulating both DNA methylation and histone H3 methylation at selected *loci* [12]. Loss of H1 resulted in DNA hypermethylation in the fungus *Ascobolus immersus* [13] and caused stochastic changes in DNA methylation in *Arabidopsis thaliana* that were linked to heritable developmental defects [14].

The complex picture of linker histone functions that emerges from the aforementioned studies is consistent with recent reports that H1s are subject to numerous post-translational modifications. The use of mass spectrometry has allowed comprehensive analysis of this phenomenon in the H1s from human, mouse and Drosophila. In animal H1s, multiple sites were shown to be modified by phosphorylation, mono-, di- and trimethylation, acetylation, formylation and ubiquitinylation [15–17]. Interestingly, in mammalian H1s, some of these modifications were also identified in the highly conserved globular domain [16].

So far, there have been no reports describing comprehensive studies on post-translational modification of plant H1s. Arabidopsis, with its limited repertoire of H1s consisting of only 3 non-allelic variants designated H1.1, H1.2 and H1.3, is a particularly well suited model for such studies. Arabidopsis H1 variants represent two distinct classes: main (H1.1 and H1.2) and stress-inducible (H1.3), a distinction that is also reproduced in a broader evolutionary context [18]. The conserved differences between H1.3 and the main H1s are located either within or very close to two characteristic binding sites on the surface of H1 globular domain (GH1). The C-terminal domain (CTD) is about 50% shorter and has a reduced overall positive charge in H1.3, compared with H1.1 and H1.2. Moreover, the N- and C-terminal domains of Arabidopsis H1.3 lack the (S/T)PXK DNA-binding motifs that occur in the corresponding domains of H1.1 and H1.2 [18]. As indicated above, Arabidopsis is a plant in which H1 has been shown to affect DNA methylation, an epigenetic mark involved in gene imprinting during gamete formation [14,19] and probably later in development, as well as during stress responses [18]. H1 has also been shown to be involved in the development of the Arabidopsis female spore mother cell [19].

Here, we have employed a novel method involving the isolation of Arabidopsis H1 histones and mass spectrometry to comprehensively characterize the type and distribution of post-translational modifications in H1s of this model flowering plant.

## Material and Methods

### Plant material

*Arabidopsis thaliana* Col-0 plants were grown in a greenhouse under natural light supplemented with sodium lamps, with a minimum day length of 16 h. The temperature was set at 21°C. Seeds were sown in soil, the pots (20 cm in diameter, 10 cm height) were placed at 6°C for 3 days without light and then transferred to the greenhouse. After 7 days the seedlings were thinned out to leave about 100 plants per pot. Four weeks after sowing, the aerial parts of plants were harvested, frozen in liquid nitrogen and stored at -80°C until required.

### Isolation of linker histones

#### Crude preparation of linker histones

Frozen Arabidopsis plants were homogenized to a powder using a high-speed knife homogenizer that had been pre-cooled with liquid nitrogen. 150 g of homogenized material were placed in a beaker and suspended in 1.5 l of 5% perchloric acid (PCA). The suspension was moved to a cold room (6°C) and further homogenized for 30 min in a rotor-stator type homogenizer (IKA, Germany) operating at maximum speed. After an additional 30-min agitation at 6°C on a magnetic stirrer, the suspension was transferred to 250-ml tubes. Insoluble debris was then pelleted by centrifuging in a fixed-angle rotor (Sorvall SLA-1000) at 15,000 × g at 4°C for 15 min. The supernatant was filtered through Miracloth (Merck KGaA, Germany) and the centrifugation repeated. This supernatant was collected and filtered as before. To precipitate proteins, trichloroacetic acid (TCA) was added to the supernatant to a final concentration of 25% and the mixture was agitated overnight at 6°C on a magnetic stirrer. The suspension was then placed in six 30-ml Corex glass centrifuge tubes (Corex, Corning, USA) and centrifuged in a swinging-bucket rotor (Sorvall HB-6, Thermo, USA) at 25,000 × g at 4°C for 30 min. The supernatant was removed, and another portion of the suspension poured into the same centrifuge tubes and centrifuged as before. To pellet TCA-precipitated proteins from the entire volume, this procedure was repeated multiple times (13 centrifugations). Because of the delicate and non-sticky character of the pelleted precipitate, the efficiency of this step was critically dependent on the geometry of the tubes, which precluded the use of those with a larger capacity. After the final supernatant had been removed, 30 ml of -20°C acetone was added to each tube and they were centrifuged at 25,000 × g at -10°C for 15 min. This acetone wash was then repeated. Following removal of the final supernatants, the tubes with histone-containing pellets were dried under vacuum and stored at -20°C.

#### Purification by ion-exchange chromatography

Histone-containing pellets were resuspended by adding 500 μl of 4.5% G buffer (100 mM phosphate buffer, 4.5% guanidine hydrochloride, pH 6.8) to each of 6 Corex tubes and sonicating in a bath-type ice-cooled BioRuptor UCD-200 sonicator (Diagenode, Belgium) for 15 min at maximum power. Sonication was performed in 30 s cycles, with the ultrasound source operating for 15 s in each cycle. The suspended pellet material was then transferred to silanized microcentrifuge tubes. For maximum recovery, the Corex tubes were rinsed twice with 500 μl lots of 4.5% G buffer and these washings were pooled with the suspension in the microcentrifuge tubes. These tubes were then sonicated again as described above to completely remove any visible fragments of pellet. Next, the tubes were shaken for 30 min at 1400 rpm at 4°C and centrifuged at 25,000 × g at 4°C for 15 min. The supernatants were collected in fresh 15 ml tube. The pellets were suspended in 250 μl of 4.5% G buffer and the tubes centrifuged again. These supernatants were pooled with those collected in the previous step. To a volume of 10.5 ml of histone-containing supernatant, 2 ml of 50% Bio-Rex 70 ion-exchange resin (Bio-Rad, USA) suspended in 4.5% G buffer were added, and the tube incubated for 4 h at 4°C with mixing by inversion. The suspension was then transferred to a 30-cm chromatographic column (10 mm diameter) and left to stand for 40 min to allow the resin to settle and form a homogenous layer. The valve at the base of the column was then opened to produce a flow of liquid at a rate of ~ 1 ml/min. Once all the liquid had soaked into the resin, it was washed by adding two consecutive 10 ml aliquots of 4.5% G buffer, followed by two 10 ml aliquots of 6% G buffer (100 mM phosphate buffer, 6% guanidine hydrochloride, pH 6.8). Due to high pI values, linker histones bind the resin even at 4.5% and remain bound at 6% guanidine hydrochloride, which enables their considerable enrichment during the chromatography step. Histones were eluted from the resin by adding two 10 ml aliquots of 10% G buffer (100 mM phosphate buffer, 10% guanidine hydrochloride, pH 6.8). The washings and eluate were then placed in dialysis tubing and dialyzed three times against 5-l volumes of 0.1% acetic acid at 6°C. The dialyzed samples were frozen in liquid nitrogen and lyophilized prior to further separation/analysis by HPLC.

#### Chromatographic separation

Linker histones were separated using an HPLC system equipped with a UV-Vis detector set to a wavelength of 220 nm. The lyophilized proteins were solubilized in 1 ml of 0.1% trifluoroacetic acid (TFA) and loaded onto a C-18 HPLC column (ACE, UK, 4.6 × 250 mm, particle size 3 μm, pore size 300Å). The column was washed for 10 min with buffer A (0.1% TFA) and eluted with a linear gradient of buffer B (90% acetonitrile, 0.1% TFA). The proportion of buffer B was ramped from 33% to 43% over 100 min. Eluate fractions were collected manually, frozen in liquid nitrogen, lyophilized and dissolved in 50 μl of deionized water for further analysis.

### Measurements with a MALDI-TOF mass spectrometer

HPLC-separated fractions were analyzed using a Bruker Ultraflex Extreme MALDI -TOF (Matrix-Assisted Laser Desorption/Ionization—Time of Flight) mass spectrometer operated in linear mode. 0.5 μl of each sample was loaded on the target plate and mixed with 0.5 μl of matrix solution (saturated synapinic acid in 50% acetonitrile and 0.1% TFA).

### Digestion of proteins

Samples of each HPLC-separated fraction were digested with four different endoproteases: trypsin, Arg-C, thermolysin (Promega, USA) and immobilized pepsin (Thermo Scientific, USA). The digestions were performed according to standard protocols provided by manufacturers.

### Analysis by Liquid Chromatography coupled with Mass Spectrometry (LC-MS)

After proteolytic digestion, the resulting peptide samples were analyzed in a nanoHPLC-MS system consisting of a nanoAcquity HPLC (Waters, USA) equipped with trap (Waters, USA, 180 μm × 20 mm, particle size 5 μm, pore size 120Å, C-18 resin) and analytical (Waters, USA, 75 μm × 250 mm, particle size 1.7 μm, pore size 120Å, C-18 resin) columns, coupled with an Orbitrap Velos mass spectrometer (Thermo Scientific, USA). Peptides were separated with a standard 0–40% gradient of acetonitrile in 0.1% formic acid. The mass spectrometer was operated in data-dependent mode with each MS scan followed by up to 5 MS/MS scans. Both MS and MS/MS spectra were acquired with an Orbitrap detector. Higher-energy Collisional Dissociation (HCD) was used for peptide fragmentation. For MS spectra the resolution of the Orbitrap was set to 30,000 and for MS/MS spectra it was set to 15,000.

### Identification of proteins and post-translational modifications

The raw data obtained by LC-MS analysis were processed with Mascot Distiller 2.4.2 (Matrix Science, UK) to obtain Mascot Generic Files (MGF) containing peptide fragmentation data. For peptide identification and the detection of post-translational modifications, Mascot server 2.4.1 was used. Several Mascot searches were performed with different settings (standard and error-tolerant searches with different combinations of post-translational modifications). Both Mascot Distiller-processed and raw spectra of potentially modified peptides were analyzed manually. MS Product from the Protein Prospector package (http://prospector.ucsf.edu) and Expert GUI [20] were used for manual assessment of data processed with Mascot Distiller. Raw data were reviewed using Thermo Xcalibur (Thermo, USA). Only modifications that could be assigned to specific positions using MS/MS spectra were considered. Masses of modifications were preferably calculated on the basis of average mass shift of multiple ions in MS/MS spectra. Since the resolution of the Orbitrap analyzer increases with a decrease in m/z, fragment ions of low mass were preferentially used to perform the calculations.

### Multiple sequence alignment and 3D modelling

Homologs of *A*. *thaliana* histone H1 variants were identified by PSI-Blast [21] searches (E-value threshold of 0.005) of the NCBI non-redundant protein sequence database. Multiple sequence alignment for GH1 domain was generated using the PCMA program [22] followed by some manual adjustments, taking into account the predicted secondary structure, hydrophobic profile of the family, conservation of important residues and the position of insertions/gaps within the 3D structure. Secondary structures were predicted with PSIPRED [23]. 3D models of the GH1 domain of H1.2 (*A*. *thaliana*) and H1.3 (*H*. *sapiens* and *M*. *musculus*) were constructed with MODELLER [24] using the closest homologs of known structure as templates [25]: *G*. *gallus* histones H5 (pdb|1hst) [26] and H1 (pdb|1ghc) [27], respectively. Side-chain rotamers were optimized using the SCWRL4 package [28]. Previously published symmetric [1] and asymmetric [2,3] models of the histone H1-nucleosome complex were obtained from the authors of the original reports and the GH1 they used was replaced by 3D model of the Arabidopsis H1.2 GH1 domain. Asymmetric models of the H1.2 GH1-nucleosome complex were superimposed onto an X-ray structure of a tetranucleosome (PDB|1zbb) [29] to derive GH1-tetranucleosome complexes.

## Results

### Isolation and analysis of Arabidopsis linker histones

**Fig 1:**
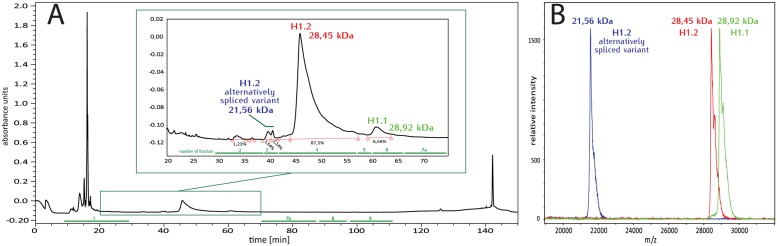
Chromatographic separation of total Arabidopsis H1. (A) Chromatogram. Masses and corresponding identification of H1 variants in different fractions are indicated on enlarged fragment of the chromatographic profile. (B) MALDI spectra of H1 variants.

Due to its small genome, Arabidopsis has a relatively low amount of histones per cell. This makes the isolation of linker histones more difficult than from plants with larger genomes, like tobacco or wheat, in which the H1s are sufficiently enriched in HClO_4_ extracts to be visualized upon SDS-PAGE. Therefore, we adopted and optimized a novel multi-step procedure for the isolation and purification of Arabidopsis linker histones, based on extraction with perchloric acid followed by ion-exchange chromatography (S1 Fig). The procedure was based on numerous preliminary experiments using both Arabidopsis and tobacco, including optimization of the HPLC gradient such that linker histones eluted between 30 and 70 minute of the run. HPLC separation of total Arabidopsis H1s obtained by this procedure revealed at least five peaks with retention times characteristic for linker histones (from 30 to 70 minutes) (Fig 1a). In addition, several strong peaks appeared at the beginning of the chromatogram and one distinctive peak was eluted with 90% acetonitrile/0.1% TFA after 145 minutes. Proteomic analyses by digestion of the proteins in solution and measurement of total protein mass by MALDI-TOF spectrometry showed that the peaks eluting in the first 20 minutes consisted predominantly of HMG (High Mobility Group)-type proteins and not H1s. We were unable to identify the substances eluting at the end of the chromatography run, indicating that they were probably non-protein in nature.

The peak detected at 47 minutes accounted for 87.5% of the total signal detected between 30 and 70 minutes of the run. A further 6.6% of this signal was represented by a peak at 61 minutes, with the remaining signal within a series of small peaks eluting between 33 and 43 minutes (Fig 1a). A total of 12 fractions were collected during each HPLC run. Fraction 1 contained material eluted up to 30 minutes and fraction 12, material eluted after 115 minutes. The remaining fractions designated 2, 3, 4, 5, 6, 7a, 7b, 8, 9 and 10, were collected during the period of H1 histone elution and immediately afterwards. Measurements with the MALDI-TOF spectrometer revealed that fractions 4 (46 min) and 6 (61 min) contained proteins of 28.45 and 28.92 kDa, respectively, which correspond to the masses of linker histone variants H1.2 (TAIR AT2g30620.1, fraction 4) and H1.1 (TAIR At1g06760.1, fraction 6). The spectra of each of these two fractions contained an additional peak with a mass about 190 Da higher than that of the main peak, which is likely to represent a histone with one or more post-translational modifications. Due to the limited resolution of the MALDI-TOF mass spectrometer, we were unable to precisely measure the mass shifts of these peaks. Fraction 3 contained a protein with a mass of 21.56 kDa, which corresponds to a second splice variant of H1.2 (TAIR At2g30620.2) (Fig 1b). Notably, the MALDI-TOF spectra of peaks containing H1 histones were broader than those of peaks containing other proteins. In addition, the top of each of the H1 peaks showed a pattern of smaller peaks, suggesting numerous post-translational modifications of the protein. The resolution of the linear mode MALDI-TOF mass spectrometer was insufficient for precise measurements of the mass differences between these small sub-peaks. Because of the weak signal, presumably caused by low abundance, we were unable to measure the masses of proteins present in fractions collected between minutes 30 and 37, including the peak representing 1.23% of the total signal (Fig 1).

### Post-translational modifications of Arabidopsis H1 variants

#### The approach

To obtain a fine map of post-translational modifications, we digested H1 variants present in all 12 HPLC fractions with four different proteases, and analyzed the resulting peptides by nano-HPLC coupled with a mass spectrometer. The enzymes used were trypsin (cleaving at K and R residues), ArgC (cleaving at R residues), thermolysin (cleaving at L, I, A, F, V and M residues) and pepsin (cleaving preferentially at F and L residues). In total, 44 HPLC MS runs were carried out. HCD in-beam fragmentation combined with measurements made using the Orbitrap analyzer enabled the recording of daughter ions with masses from 100 Da. The use of the Orbitrap permitted high resolution measurements of both the parent and daughter ions with an accuracy of 0.01 Daltons. Raw data acquired during experiments are available at http://h1mod.arabidopsis.pl/ and under DOI:10.5281/zenodo.35667.

The collected data enabled the identification of 490 and 558 unique peptides of H1.1 and H1.2, respectively, and 10 peptides of H1.3, the latter occurring at a much lower level. As shown by MALDI-TOF analysis (Fig 1), particular H1 variants were eluted at different times. However, despite using a very flat gradient, the tails of the peaks did not reach the base-line (marked in red on Fig 1), most likely because of the high similarity of the variants and the presence of different post-translational modifications altering the retention times. Because of the much higher sensitivity of identification with the LC-MS system compared to whole protein measurements using MALDI MS, peptides of all three H1 variants were detected in all analyzed chromatographic fractions. Data obtained from analyses of the separate fractions were therefore pooled before identifying the post-translational modifications (PTMs). This yielded four datasets representing the analyses with the four different proteases. The largest amount of peptides was detected in digests with trypsin, followed by those with ArgC, thermolysin and pepsin. Trypsin digestion led to the identification of the highest number of PTMs. In addition to H1, we detected a number of other proteins, including various HMG proteins (S1 Table). The identification score of these proteins was at least 2.5-times lower than that of the H1s.

From the obtained data, we were able to identify numerous post-translational modifications of the main H1 variants (H1.1 and H1.2). Peptides found during the analyses covered the complete sequences of these two proteins. Despite finding peptides covering 88% of the H1.3 sequence, the probability of detecting post-translational modifications of H1.3 was very low because the number of these peptides was many times lower than the number of peptides of H1.2 and H1.1. Therefore, we were unable to reliably ascribe post-translational modifications to H1.3.

#### Type and distribution of post-translational modifications in Arabidopsis H1s

**Fig 2:**
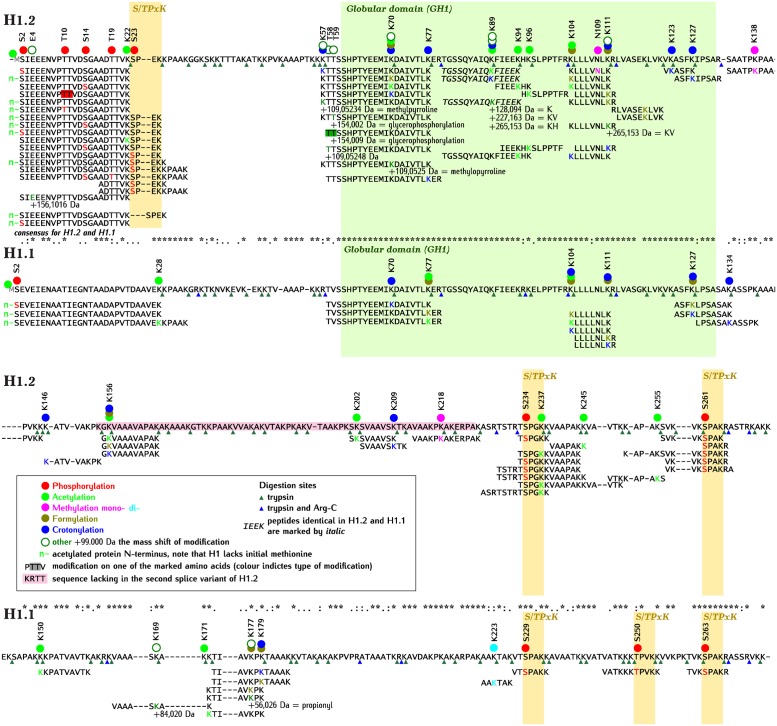
Alignment of amino acid sequences of Arabidopsis H1.2 and H1.1 with identified post-translational modifications. The peptides with modifications identified by mass spectrometry are shown under the corresponding full sequence. Circles over the full sequence mark amino acids, with color filling the circle indicating the type of modification. For modifications marked “other” (white circle with dark green contour), the mass of modification in Da (eg. +99.000 Da) and the name of the possible chemical compound (eg. glycerophosphorylation) are indicated. The symbols: K, KV and KH correspond to lysine, lysine-valine and lysine-histidine, respectively. The color of letters corresponding to modified amino acids in the peptides corresponds to the type of modification, as shown for circles. Fragments of peptides identical in H1.2 and H1.1 are marked by italics. The sequences corresponding to globular domain (GH1) and S/TPxK motives are shaded in green and yellow, respectively. Sequence absent in a second splice variant of H1.2 is shaded in pink. Digestion sites by trypsin and trypsin and Arg-C proteases are marked by green and blue triangles, respectively. Green “n-” denotes acetylated protein N-terminus. Note that H1.2 and H1.1 lack initial methionine (amino acid 1) marked by grey “M” in the full sequence.

Our analyses revealed 30 amino acids of H1.2 and 17 amino acids of H1.1 that were post-translationally modified. One modified lysine of H1.2, marked as K89 on Fig 2, was found in a peptide that was identical in both H1.2 and H1.1. Some amino acids were targets of several (up to four) different modifications. Many of the detected modifications, such as formylation, acetylation, methylation and crotonylation of lysine, phosphorylation of serine and threonine, and acetylation of the N-terminus, are typical for histones. However, we also detected modifications that have not previously been seen in histones (see below). The peptides of H1.1 and H1.2 carrying post-translational modifications, are listed in Fig 2, which also shows an alignment of the amino acid sequences of these H1 variants.

#### N-terminal domain

All three Arabidopsis H1 variants, like the majority of histones, lack the N-terminal methionine residues encoded by their corresponding genes. We detected numerous peptides representing the N-terminal domain starting with a serine, but none started with a methionine (this missing N-terminal methionine is however still taken into account when numbering the amino acids). The N-terminal domain of both of the two main Arabidopsis H1s is comprised of ~ 60 amino acids. In contrast to the adjacent globular domain (GH1), its sequence is not highly conserved (Fig 2). In both H1.1 and H1.2, it consists of an initial fragment of acidic character (amino acids 1–28 in H1.1 and 1–25 in H1.2) followed by an internal fragment containing numerous basic residues. The N-terminal domain of H1.3, which consists of ~ 20 amino acids, contains 8 basic- and only 2 acidic residues.

Phosphorylation was found at five positions within the acidic part of the H1.2 N-terminal domain, namely on serine 2 (the first amino acid of the mature protein), serine 14 and serine 23, and on threonines 10 and 19. In the N-terminal domain of H1.1, only S2 was phosphorylated. The initial serine of H1 is highly conserved, being present in H1.2, H1.1 and in most H1 variants of mammals and Drosophila but, surprisingly, not in H1.3 of *A*. *thaliana*. In both H1.1 and H1.2, the N-terminal domain was acetylated on the first lysine (K22 in H1.2 and K29 in H1.1) (Fig 2). In the N-terminal domain of H1.2 the glutamic acid residue at position 4 was also modified. The mass of this modification was 156.10 Da which corresponds to arginine. While we found only a single peptide of H1.2 with this modification, its identification is fairly certain since it was described by ions of the b series and by numerous internal ions.

We did not detect any modifications in the middle part of the basic fragment of the H1 N-terminal domain. However, at the boundary of this fragment and the globular domain in the H1.2 variant, we identified crotonylation of K57. Independently, in the same variant, on the neighboring residues K57 and T58 we detected modifications with masses of 109.05234 Da (a single MS/MS spectrum) and 109.05248 Da (4 MS/MS spectra), respectively, which correspond to methylpyrroline (109.052764 Da), a modification described in certain Archaea [30] (Fig 2). There is no combination of C, O, N and H atoms, as an alternative to methylpyrroline, that differs from the measured mass by less than 0.01 Da. However, we cannot exclude the possibility that the modification corresponds to another compound that shares its empirical formula with methylpyrroline. On neighboring T59 another modification was detected with a mass of 154.009 Da, corresponding to phosphoglycerol (Fig 2). A modification with a very similar mass (154.002 Da) was found in a peptide with the same sequence.

#### Globular domain

The globular domain (GH1), which comprises about 70 amino acids, differs in only 5 positions between the two main variants. It was therefore impossible to determine the origin of some of the identified peptides (marked by italics under the sequence of H1.2 in Fig 2). GH1 was found to be modified by acetylation, formylation and crotonylation of lysine residues. We also identified methylation of asparagine. A modification with a mass shift corresponding to methylpyrolline (109.025 Da) was detected on K70 in an N-terminal fragment of GH1. On K89 located in the middle part of GH1, in a peptide that could be derived from either of the two main H1 variants, we found three different modifications: one with the mass of 128.094 Da and two others with masses of 227.163 Da and 265.153 Da, respectively. The masses of these modifications equal the mass of a single lysine (K), and of lysine-valine (KV) and lysine-histidine (KH) dipeptides, respectively. This residue K89 was also subject to acetylation and crotonylation. The modifications that could be unambiguously ascribed to the GH1 of H1.1 were acetylation (K77 and K104), formylation (K77, K104, K111, K127) and crotonylation (K70, K104, K111, K127) (Fig 2).

#### C-terminal domain

The lysine-rich C-terminal domain, consisting of ~ 140 amino acids, is the longest region of each of the two main Arabidopsis H1variants. In both H1.1 and H1.2, it contains S/TPxK motifs known to bind cyclin-dependent kinases (CDKs). All of these motifs were found to be targets of phosphorylation. In one S/TPxK motif in H1.2, the acetylation of lysine was also detected. Since CDKs have been shown to have a high preference for lysine or arginine as the n+2 or n+3 residue (n being the position of phosphoacceptor serine or threonine in S/TPxK) [31], the acetylation of lysine within this motif is likely to strongly interfere with CDKs phosphokinase activity.

Two more acetylated lysines were found between two S/TPxK motifs near the H1.2 C-terminus. In summary, lysines within the H1.2 C-terminal domain were found to be methylated, acetylated, formylated and crotonylated, and those of the H1.1 C-terminal domain, dimethylated, acetylated, formylated, crotonylated and propionylated. An unidentified modification with a mass of 84.02 Da also occurred on one of the H1.1 lysines (Fig 2).

### Location of GH1 post-translational modifications in the chromatosome structure

**Fig 3:**
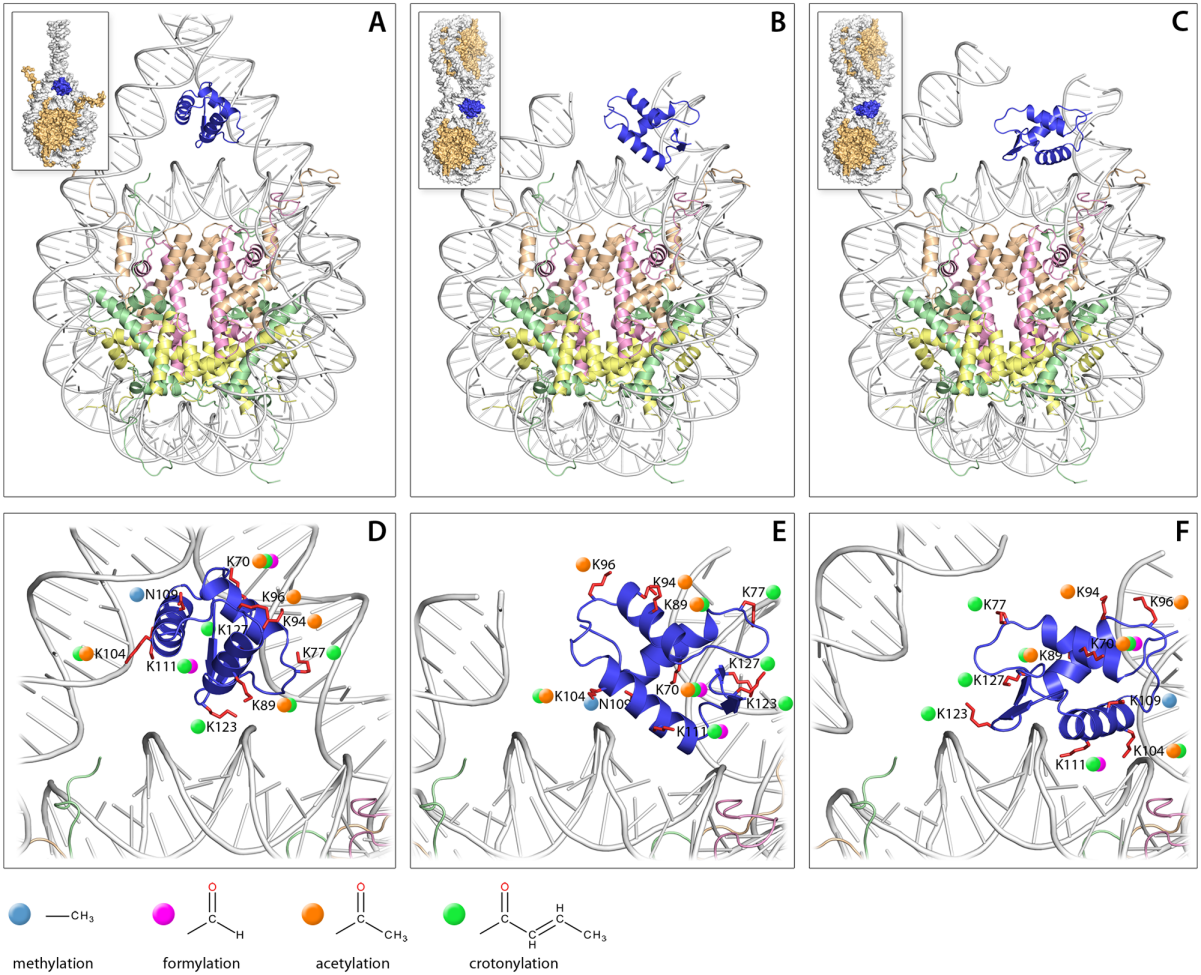
3D models of the GH1 domain of Arabidopsis H1.2 in complex with a nucleosome. (A) symmetric model of GH1-nucleosome complex from Syed et. al. [1], (B) asymmetric model from Zhou et. al. [2], (C) asymmetric model from Song et. al. [3]. The presented structures correspond to the respective H1-nucleosome models obtained from the authors, with the original GH1 replaced by the 3D model of the Arabidopsis H1.2 GH1 (blue). Schematic representations of GH1-mononucleosome and GH1-dinucleosome complexes are shown in the upper left corners. (D-F) Enlargement of GH1 binding with residues targeted by post-translational modifications shown in red. The identified modifications are denoted by colored dots: methylation—magenta; formylation—olive; acetylation—green; crotonylation—blue. The models in D, E and F are shown in the same orientation as those in A, B and C, respectively.

In order to map the locations of PTMs identified in the H1 globular domain onto its tertiary structure, we constructed a 3D model of *Arabidopsis* H1.2 GH1 using a crystal structure of linker histone H5 (PDB|1hst) [26] as template. The model revealed that the identified post-translational modifications encompassing mono-methylation of N109 and various formylations, acetylations and crotonylations of the side chains of several lysine residues are dispersed over the entire GH1 surface, including DNA binding sites I, II and III [e.g. K77(II), K104(I), K111(I), K123(III), K127(II)] (Fig 3). We also analyzed the locations of these PTMs in the context of the nucleosome, using the three most recent models of the histone H1-nucleosome complex (Fig 3), including (i) a symmetric model of GH1 interacting with the DNA minor groove, with GH1 located at the center of the nucleosome and contacting both linker DNAs [1] and (ii) two models where GH1 bridges the nucleosome core and one entering/exiting linker DNA asymmetrically [2,3]. The selected models represent different possible modes of GH1 binding: in the symmetric model the α3 helix of GH1 interacts with the major groove of linker DNA, while in the asymmetric models it faces the major groove of nucleosomal DNA near the dyad axis (in addition, the α3 helix is oriented in opposite directions in the two types of asymmetric model). We found that regardless of the model employed, most of the residues (lysines) identified as post-translationally modified in Arabidopsis GH1 seem to be involved in DNA binding, especially when considering the higher-order chromatin structure. While none of the above models is favored as optimal for GH1 binding based on our analysis, it is clear that the identified PTMs interfere, most likely negatively, with specific binding of GH1 to the nucleosome, by decreasing the strength of its interactions with the charged phosphate groups of DNA.

## Discussion

The goal of this study was an unbiased comprehensive characterization of the qualitative profile of post-translational modifications of plant linker histones. Since Arabidopsis H1s –an ideal subject for such analysis—are difficult to isolate in pure form, we adopted a novel extraction method and used HPLC to separate the three different variants. Unexpectedly, given the comparable levels of H1.1 and H1.2 transcripts detected by RT-PCR [14,18], we found strong quantitative dominance of H1.2 over H1.1 protein, consistent with the mostly post-transcriptional regulation of the ratio of these two main Arabidopsis H1 variants. The evolutionarily conserved, in flowering plants, stress-induced H1.3 variant [32,33] was also detected, albeit at a very low level. We estimate that even upon up-regulation under conditions of environmental stress, H1.3 represents < 1% of total H1 protein in Arabidopsis (unpublished results). In addition, we found that a splice variant of H1.2, that is 70 amino acids shorter than the full-size protein, is also synthesized and represents ~ 2.7% of the total Arabidopsis H1 protein. The sensitivity of the Orbitrap mass spectrometer enables high quality identification of proteins representing even a very small fraction of the sample. Therefore, while particular H1 variants were identified on MALDI spectra as major proteins within their respective HPLC peaks, indicating good chromatographic separation, we could still observe peptides originating from other variants in these peaks, when the much more sensitive bottom-up approach (LC-MS) was used. Thus, in the fraction in which most of the H1.2 was eluted, peptides of H1.1 were also detected and *vice versa*. It should be noted that complete separation of two sister-proteins with properties as similar as the two major Arabidopsis H1 variants is very difficult, and even more so because both are subject to various post-translational modifications that affect their retention time on the HPLC column.

Mass spectrometry has become the prime tool for the detection of known and the identification of novel PTMs [34]. Most published data on post-translational modifications of H1 were obtained using spectrometers equipped with TOF analyzers [15,17,35], which are less precise than FT-ICR or Orbitrap analyzers. The use of the latter allows the discrimination of PTMs with very similar masses. For example, two modifications that can only be distinguished by accurate mass measurements are lysine dimethylation and lysine formylation, which differ in mass by 0.036 Da [36]. All data in this work were obtained using a ‘high-high’ strategy where both MS and MS/MS spectra were recorded with the high resolution Orbitrap analyzer. While the detection by MS of well characterized PTMs typically found in histones is fairly unambiguous, the identification of novel modifications requires independent experimental verification. Our major goal in this work was to reveal the overall extent and variety of PTMs present in Arabidopsis H1 variants, as well as to map the location of these modifications within H1 molecules. The identification of the novel PTMs was based on MS and other available data; extended confirmation procedures are beyond the scope of this study and will be a part of future investigations.

Overall, we found that Arabidopsis H1 histones are the target of numerous post-translational modifications that also occur in H1s of animal lineages, including phosphorylation, acetylation, mono- and dimethylation, formylation, crotonylation and propionylation. However, we also identified some modifications that have not been previously described in histones.

### Phosphorylation

Phosphorylation is the most frequently described modification of linker histones [15–17,37]. We identified seven phosphorylated serines and/or threonines in H1.2 and four in H1.1. Similarly, to the H1s of birds, mammals and Drosophila [16,35,37], the N-terminal serines are phosphorylated in both H1.1 and H1.2. The fact that serine is the N-terminal amino acid of most H1 variants (after removal of the initial methionine) in such evolutionarily distant lineages as Arabidopsis and vertebrate and invertebrate animals, suggests functional importance of this feature. As in animal homologs, phosphorylation in Arabidopsis H1s occurs both within and outside the S/TPxK motifs. While in vertebrates, one or two phosphorylated sites were reported between amino acids 2–23 and one or two at the boundary of the N-terminal domain and GH1 [16,35], the Arabidopsis H1.2 variant has four phosphorylated sites between amino acids 2–23 (one at S23 within the S/TPxK motif) and none at the boundary of the N-terminal domain and GH1. This is closer to the pattern detected in Drosophila H1 [37]. Despite these differences in distribution, the N-terminal domains of H1s from all lineages analyzed so far seem to be phosphorylated at a similar number of sites. Interestingly, unlike Drosophila H1 [16,35] and similarly to human H1s [16], no phosphorylation was found within the globular domain of Arabidopsis H1s.

### Acetylation

Similarly, to phosphorylation, the acetylated N-terminal serine and numerous acetylated lysine residues are present in all H1s in which PTMs have been studied. Consistent with this pattern, we found lysine acetylation in ten positions in H1.2, five positions in H1.1 and one position that could be in both of these variants. Unlike in Drosophila and similarly to human H1s [16,17,37], lysine acetylation was particularly frequent in the Arabidopsis H1 globular domains. As in previous studies, we did not detect acetylated arginine residues in Arabidopsis H1s.

### Propionylation

Lysine propionylation was recently reported in an H1 isolated from mouse brain [38] and in histone H3 from a mammalian cell line [39]. It is probably added and removed by the same enzymes that are responsible for histone acetylation and deacetylation, except that propionyl-CoA is employed instead of acetyl-CoA [39,40]. Together with the recently discovered lysine butyrylation [41], succinylation and malonylation [42], this indicates a tight connection between histone modification status and cell metabolism. Here, we detected a modification with a mass of 56.026 Da on one lysine residue in the C-terminal domain of Arabidopsis H1.1, which corresponds to propionylation (the difference in mass is < 0.0003 Da).

### Methylation, dimethylation and formylation

We detected mono-methylation at two lysines located in the C-terminal domain of H1.2, and one methylated asparagine located in the H1.2 globular domain. Only a few methylated amino acids have been found in animal H1s, and methylated asparagine has never before been reported in histones [16,17,37]. Here, asparagine methylation was found in three MS/MS spectra, which contained all peaks of the b and y series. This modification was mapped by numerous internal fragments, which strongly supports its identification.

Di-methylated lysine has often been found in histones. Its mass is very close to that of formylated lysine, the occurrence of which in histones was recognized only recently [43], and which turns out to be one of the most common H1 modifications [36]. In most previous reports, formylation and dimethylation of H1 were not distinguished. As mentioned above, these modifications can only be differentiated using high-resolution spectrometers like FT-ICR or the Orbitrap. Therefore, our data can only be compared with those of Wisniewski and coworkers [16,36]. While we found only one di-methylated lysine in the C-terminal domain of H1.1, lysine formylation was much more frequent. It occurred at four positions in H1.2 (three in the globular and one in the C-terminal domain) and at five positions in H1.1 (three in the globular and two in the C-terminal domain). Formylation often occurred at positions in which other modifications were observed, particularly acetylation and crotonylation (Fig 3). The occurrence of formylated lysines in the GH1 and C-terminal domains of Arabidopsis H1s resembles their distribution in mammalian H1s [36].

### Crotonylation

Lysine crotonylation was first described in core histones of yeast, mammals and Drosophila by Tan and colleagues in 2011 [44]. This modification is particularly frequent in Arabidopsis H1s, since we found nine crotonylated lysines in H1.2 and six in H1.1 (one of the identified peptides with crotonylation could have been derived from either H1.2 or H1.1). Lysine crotonylation occurred in both the globular and C-terminal domains of these H1s. It has been shown that in HeLa cells crotonyl-CoA stimulates transcription through core histone crotonylation catalyzed by HAT (histone acetyl transferase). The fact that HAT also acetylates histones using acetyl-CoA as the cofactor, again suggests a strong link between chromatin status and the metabolic state of the cell, in this case determined by competition between different HAT cofactors [45]. It will be interesting to determine whether crotonylation of Arabidopsis H1s is also involved in the stimulation of transcription.

### Novel PTMs

Peptide sequence and mass shifts marked with green letters in Fig 2 are modifications (with their masses indicated) that have not previously been described in histones of any organism. We detected these novel modifications mostly in H1.2, probably because it was present at a much higher concentration than the other variants in the studied samples.

### Putative modifications by attachment of peptides to lysine

On K89 within a peptide derived from GH1, we detected a modification with a mass corresponding to lysine within a polypeptide chain (128.094 Da). At the same position of GH1 we also detected peptides with mass shifts corresponding to the combined masses of lysine and valine (227.163 Da) and lysine and histidine (265.153 Da). This suggests that K89 was modified by the attachment of some branching polypeptides, which might have become fragmented during sample preparation. Such branching requires the formation of an isopeptide bond between the ε-amino group of lysine and either the free carboxyl terminus of another protein (as in the case of ubiquitination and sumoylation), or side chain carboxyl groups of glutamate or aspartate. If the observed mass shifts do indeed represent the addition of the aforementioned amino acids, one plausible explanation would be modification by a protein with a C-terminal lysine. While well supported by data, these unusual modifications need to be further investigated using independent methods.

### Putative modification with the mass of phosphoglycerol

On T59 of H1.2 we identified a mass shift of 154.002 Da, which is characteristic for phosphoglycerol, a modification found in the pilin protein of Neisseria [46]. We detected *y*-ions mapping the modification and almost the entire sequence of the peptide. We could also detect a neutral loss of 63.96 Da, mostly in the b-ion series. Since we were unable to assign the mass of this neutral loss to any part of the glycerophosphorylation, it is possible that this unusual modification has a different chemical nature.

### Putative modification with the mass of arginine (156.1016 Da)

The observed mass change on I3 of H1.2 could be accounted for by several possible combinations of atoms besides arginine. After discarding unrealistic combinations with four or more N atoms or a high number of C, O or N and only a few H atoms, the most plausible combination is C_8_H_14_O_2_N. The putative molecule described by this formula differs from the measured mass by only 0.0003 Da. The second closest hit is arginine with a mass difference of 0.001 Da. The formula C_8_H_14_O_2_N with added hydrogen was used to search the Reaxys database (www.reaxys.com) and was found to correspond to 13 different compounds known to occur in living organisms. Thus, the identification of the true nature of this modification awaits further studies.

### Putative methylpyrolline

On K57 and K70 of H1.2, we found a modification producing a mass shift of 109.052 Da. While such a shift could be due to the addition of different compounds, of all the combinations of H, C, N and O atoms, it is most similar to methylpyrolline (4-methylpyrroline-5-carboxylate), a modification found in certain Archaea [47].

### Putative modification with a mass of 84.020 Da

Another unknown modification with a mass of 84.020 Da was detected on K169 in the C-terminal domain of H1.1. We have been unable to identify a plausible combination of atoms to account for this mass shift. It is possible that the modification is not on the ε-amino group, but includes some changes to the lysine side chain.

### Modification ‘hot spots’

At several positions in the H1 sequence, a number of different modifications were detected. Most of these modification ‘hot spots’ are lysine residues within the globular domain (K70, K89, K111), and one lysine in the C-terminal domain of H1.2 (K156). There are also numerous regions where different modifications can occur in close proximity. These sites are likely to be critical for H1 function and for the regulation of different processes that depend on its interactions in chromatin.

## Conclusions

**Fig 4:**
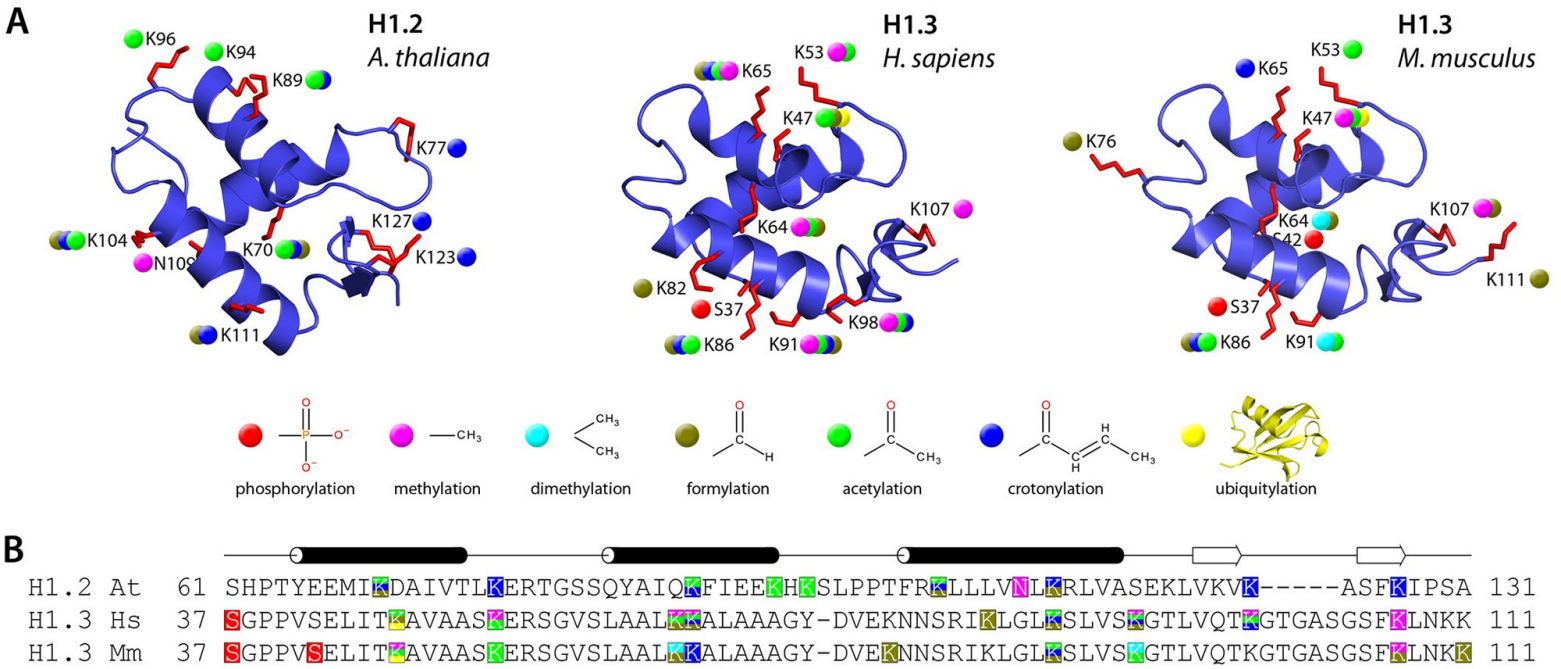
Comparison of post-translational modifications in the GH1 domains of Arabidopsis H1.2, and human and mouse H1.3. (A) 3D models of the GH1 domains of *A*. *thaliana* H1.2, *H*. *sapiens* H1.3 and *M*. *musculus* H1.3. Residues subject to post-translational modification in Arabidopsis H1.2 identified in this study and those reported for human and mouse H1.3 by Wisniewski et. al. [16,36] and Tan et. al. [44] are shown in red. Colored dots denote the modifications according to the key. The models are shown in the same orientation as in Fig 3E. (B) Black cylinders and whiter arrows represent α-helices and β-turn, respectively. Multiple sequence alignment of the GH1 domains of Arabidopsis, human and mouse H1s. Residues modified post-translationally are highlighted using the same color scheme as in A.

Our analyses have revealed that the H1 linker histones of plants, here represented by the model flowering plant *Arabidopsis thaliana*, like their animal homologs, are targeted by a highly diverse array of post-translational modifications. Despite over 1 billion years of independent evolution, the post-translational modifications of H1s from flowering plants and mammals seem to share several common patterns. As shown in Fig 4, there is a remarkable similarity between the location of modified amino acids in the conserved globular domains of Arabidopsis and mammalian H1s, including those comprising specific GH1-DNA binding sites. Notably, all modified residues are located on the surface of GH1. This suggests that post-translational modifications of these amino acids, which become transiently exposed to the external environment due to dynamic on and off binding of H1 to nucleosomes, may be universally used to control the strength of the GH1-nucleosome interactions. The two main Arabidopsis H1s and mammalian H1s show similar distributions of modifications in the N-terminal domain. These include a phosphorylated terminal serine, several phosphorylated serines and threonines and acetylated lysines in the initial acidic fragment, the lack of modifications in the adjacent more basic fragment of this domain, plus numerous modifications of the short fragment at the N-terminal domain-GH1 border (Table 1). However, a surprising difference between Arabidopsis and mammalian H1s identified here concerns the C-terminal fragment directly adjacent to GH1, which is known to be involved in formation of the linker DNA stem structure and is thought to stabilize folding into higher-order chromatin fibers. This fragment is targeted by numerous modifications in Arabidopsis H1s, but not in their mammalian homologs. The functional significance of this difference remains to be established. In contrast, in both Arabidopsis and mammalian H1s the more distant fragments of the C-terminal domain are similarly targeted by numerous post-translational modifications, including multiple phosphorylations, both within and outside the S/TPxK sites (Table 1). Albeit it is difficult to estimate the frequency of different PTMs based solely on our data, we consider it likely that the phosphorylation of S14, S23 and S1 are very abundant in Arabidopsis H1s, as we find numerous peptides with strong signal for this PTMs.

**Table 1:** Comparison of post-translational modifications present in different regions of Arabidopsis and mammalian H1s.

Region	Position		Arabidopsis	Human	Comments
	A. th. H1.2	Human H1.3			
Initial serine	2	2	phospho S, acetylation of N-term	phospho S, acetylation of N-term	
N-terminal, acidic region	3–24	3–22	phospho S and T (4 sites in H1.2), acetyl K (one site)	phosphorylation, acetyl K	usually contains S/TPxK motiff (Ath H1.2, mammals) which are phosphorylated, phosphorylated also outside S/TPxK
N-terminal, basic region	25–56	23–32			
GH1-border	57–63	33–40	crotonyl K, 2 unknown modifications	methyl. (2 sites), crotonyl. (2 sites), formyl., acetyl., phospho.	
GH1	61–130	37–109	numerous modifications: acetyl., crotonyl., formyl., methyl., unknown mod.	numerous sites: acetyl., crotonyl., formyl., methyl., ubiquitin.	
C-terminal, stem-forming region	131–150	110–130	crotonyl K, methyl K, acetyl K		
The rest of C-terminal	151–273	131–221	phospho. (in 2 S/TPxK sites), multiple sites: acetyl., crotonyl., formyl., single methyl., dimethyl.	multiple sites of phospho T/S (inside and outside of S/TPxK), acetyl K, methyl K, crotonyl K	

The above comparison shows that, with the exception of the C-terminal domain fragment involved in generation of the linker DNA stem structure, both the GH1 domain and most of the unstructured N- and C-terminal domains of H1s in flowering plants and mammals are subject to similarly distributed and comparably diverse types of post-translational modifications, suggesting their conserved functional significance and equally complex biological roles. There is a growing consensus that PTMs of histones function as part of the general mechanism linking chromatin activities with the metabolic status of the cell. It is thus possible that the occurrence in plant H1s of modifications that are so far unknown in animal histones and therefore not yet characterized, may reflect some basic differences between the metabolic systems of plants and animals.

## Supporting Information

S1 Fig Flow chart depicting major steps during the isolation of Arabidopsis H1 histones. Conditions for each step are indicated on the scheme. Consult Material and Methods for more details.
(PDF)

S1 Table Proteins identified in analyzed samples obtained with the use of different proteases. Mascot scores and number of identified peptides (in brackets) are shown for each protease. Mascot searches were performed without considering post-translational modifications.
(PDF)
