# Supplementary material for: Histone H1 Variants in Arabidopsis Are Subject to Numerous Post-Translational Modifications, Both Conserved and Previously Unknown in Histones, Suggesting Complex Functions of H1 in Plants
Source: PLoS One. 2016 Jan 28;11(1):e0147908. doi: 10.1371/journal.pone.0147908 (PMC4731575; doi:10.1371/journal.pone.0147908)
Supplement: S1 Fig — Conditions for each step are indicated on the scheme. Consult Material and Methods for more details. (PDF) [file pone.0147908.s001.pdf]

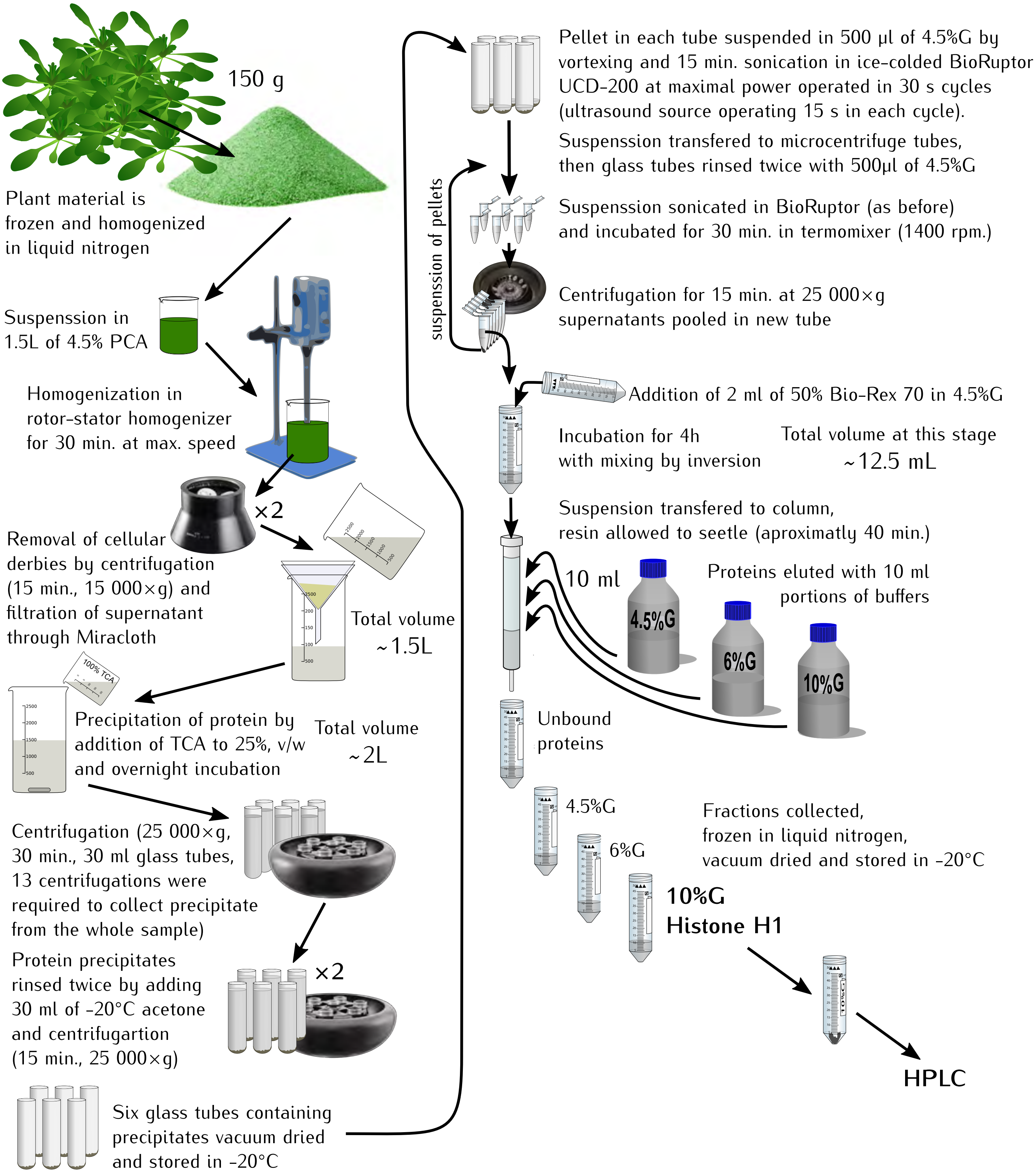
150 g
Plant material is frozen and homogenized in liquid nitrogen
Suspenssion in 1.5L of 4.5% PCA
Homogenization in rotor-stator homogenizer for 30 min. at max. speed
×2
Removal of cellular derbies by centrifugation (15 min., 15 000×g) and filtration of supernatant through Miracloth
Total volume ~1.5L
100% TCA
Precipitation of protein by addition of TCA to 25%, v/w and overnight incubation
Total volume ~2L
Centrifugation (25 000×g, 30 min., 30 ml glass tubes, 13 centrifugations were required to collect precipitate from the whole sample)
Protein precipitates rinsed twice by adding 30 ml of -20°C acetone and centrifugartion (15 min., 25 000×g)
×2
Six glass tubes containing precipitates vacuum dried and stored in -20°C
Pellet in each tube suspended in 500 µl of 4.5%G by vortexing and 15 min. sonication in ice-colded BioRuptor UCD-200 at maximal power operated in 30 s cycles (ultrasound source operating 15 s in each cycle).
Suspenssion transfered to microcentrifuge tubes, then glass tubes rinsed twice with 500µl of 4.5%G
suspenssion of pellets
Suspenssion sonicated in BioRuptor (as before) and incubated for 30 min. in termomixer (1400 rpm.)
Centrifugation for 15 min. at 25 000×g supernatants pooled in new tube
Addition of 2 ml of 50% Bio-Rex 70 in 4.5%G
Incubation for 4h with mixing by inversion
Total volume at this stage ~12.5 mL
Suspension transfered to column, resin allowed to seetle (aproximatly 40 min.)
10 ml
Proteins eluted with 10 ml portions of buffers
4.5%G
6%G
10%G
Unbound proteins
4.5%G
6%G
Fractions collected, frozen in liquid nitrogen, vacuum dried and stored in -20°C
10%G
Histone H1
HPLC
