# Supplementary material for: Histone H1 Variants in Arabidopsis Are Subject to Numerous Post-Translational Modifications, Both Conserved and Previously Unknown in Histones, Suggesting Complex Functions of H1 in Plants
Source: PLoS One. 2016 Jan 28;11(1):e0147908. doi: 10.1371/journal.pone.0147908 (PMC4731575; doi:10.1371/journal.pone.0147908)
Supplement: S1 Table — Mascot scores and number of identified peptides (in brackets) are shown for each protease. Mascot searches were performed without considering post-translational modifications. (PDF) [file pone.0147908.s002.pdf]

Supporting Information Table 1. Proteins identified in analyzed samples obtained with the use of different proteases. Mascot scores and number of identified peptides (in bracets) are shown for each protease. Mascot searches were performed without considering post-translational modifications.

nr	TAIR identifier	Name	Trypsin	Arg-C	Thermolysin	Pepsin
1	AT2G30620.1	H1.2 | winged-helix DNA-binding transcripti	30756(219)	37235(199)	2829(124)	1844(16)
2	AT2G30620.2	H1.2a | winged-helix DNA-binding transcript	30345(192)	35853(185)	1746(97)	1844(16)
3	AT1G06760.1	H1.1 | winged-helix DNA-binding transcriptio	16699(139)	26121(101)	2539(68)	884(9)
4	AT1G48610.1	| AT hook motif-containing protein | chr1:	11036(104)	9336(81)	1056(42)	
5	AT1G48610.2	| AT hook motif-containing protein | chr1:	10544(99)	8421(75)	1056(39)	
6	AT1G28290.1	AGP31 | arabinogalactan protein 31 | chr1:	9869(79)	4002(45)	519(18)	154(9)
7	AT1G28290.2	AGP31 | arabinogalactan protein 31 | chr1:	9869(79)	4002(45)	519(18)	154(9)
8	AT1G76180.2	ERD14 | Dehydrin family protein | chr1:28	5503(43)	189(7)	923(18)	217(3)
9	AT1G76180.1	ERD14 | Dehydrin family protein | chr1:28	5503(43)	189(7)	923(18)	217(3)
10	AT1G20440.1	COR47, RD17, AtCOR47 | cold-regulated 47	4287(21)	291(4)	516(11)	
11	AT1G13930.3	| Involved in response to salt stress. Knocko	4043(17)	1647(10)	466(14)	56(2)
12	AT1G13930.1	| Involved in response to salt stress. Knocko	4043(17)	1647(10)	466(14)	56(2)
13	AT1G13930.2	| Involved in response to salt stress. Knocko	4043(17)	1647(10)	466(14)	56(2)
14	AT3G53740.2	| Ribosomal protein L36e family protein | c	1819(22)	3150(23)	676(11)	557(8)
15	AT3G53740.3	| Ribosomal protein L36e family protein | c	1819(22)	3150(23)	676(11)	557(8)
16	AT3G53740.4	| Ribosomal protein L36e family protein | c	1819(22)	3150(23)	676(11)	557(8)
17	AT1G62480.1	| Vacuolar calcium-binding protein-related	3138(12)	189(2)	902(17)	
18	AT5G02450.1	| Ribosomal protein L36e family protein |	2371(29)	2887(23)	811(14)	409(5)
19	AT3G51880.1	HMGB1, NFD1 | high mobility group B1 | c	2585(29)	1067(17)	518(14)	
20	AT3G51880.3	HMGB1, NFD1 | high mobility group B1 | c	2585(29)	1067(17)	518(14)	
21	AT1G20696.3	HMGB3, NFD3, NFD03 | high mobility group	2367(30)	947(25)	1619(16)	
22	AT1G20696.1	HMGB3, NFD3, NFD03 | high mobility group	2367(30)	947(25)	1619(16)	
23	AT1G20693.3	HMGB2, HMG BETA 1, NFD2, NFD02 | high	2247(22)	1217(24)	814(16)	81(5)
24	AT1G20693.1	HMGB2, HMG BETA 1, NFD2, NFD02 | high	2247(22)	1217(24)	814(16)	81(5)
25	AT1G20693.2	HMGB2, HMG BETA 1, NFD2, NFD02 | high	2247(22)	1217(24)	814(16)	81(5)
26	AT3G51880.2	HMGB1, NFD1 | high mobility group B1 | c	2163(26)	1067(17)	518(14)	
27	AT3G51880.4	HMGB1 | high mobility group B1 | chr3:19	2163(26)	1067(17)	518(14)	
28	AT1G20696.2	HMGB3, NFD3, NFD03 | high mobility group	2161(26)	947(25)	1160(14)	
29	AT4G20260.1	ATPCAP1, PCAP1 | plasma-membrane associate	2098(26)		594(20)	
30	AT4G20260.3	ATPCAP1, PCAP1 | plasma-membrane associate	2098(26)		594(20)	
31	AT4G20260.2	ATPCAP1, PCAP1 | plasma-membrane associate	2098(26)		594(20)	
32	AT4G20260.4	ATPCAP1, PCAP1 | plasma-membrane associate	2098(26)		594(20)	
33	AT3G53740.1	| Ribosomal protein L36e family protein | c	1819(22)	2079(19)	676(10)	557(8)
34	AT1G15340.1	MBD10 | methyl-CPG-binding domain 10 | c	2056(24)	108(3)	907(16)	
35	AT1G15340.2	MBD10 | methyl-CPG-binding domain 10 | c	1835(23)	108(2)	903(14)	
36	AT2G37600.1	| Ribosomal protein L36e family protein | c	661(18)	1657(30)	50(7)	
37	AT2G37600.2	| Ribosomal protein L36e family protein | c	661(18)	1657(30)	50(7)	
38	AT2G20260.1	PSAE-2 | photosystem I subunit E-2 | chr2	948(11)	1550(10)	320(7)	300(3)
39	AT3G49010.3	ATBBC1, BBC1, RSU2 | breast basic conserv	1538(14)	607(6)	58(6)	358(3)
40	AT3G49010.1	ATBBC1, BBC1, RSU2 | breast basic conserv	1538(14)	607(6)	58(6)	358(3)
41	AT3G49010.2	ATBBC1, BBC1, RSU2 | breast basic conserv	1538(14)	607(6)	58(6)	358(3)
42	AT1G08830.2	CSD1 | copper/zinc superoxide dismutase 1	1427(8)	1117(6)	992(12)	
43	AT1G08830.1	CSD1 | copper/zinc superoxide dismutase 1	1427(8)	1117(6)	992(12)	
44	AT5G25610.1	RD22, ATRD22 | BURP domain-containing pro	1403(6)	262(4)	231(2)	
45	AT3G49010.4	ATBBC1, BBC1, RSU2 | breast basic conserv	1380(13)	376(5)	58(5)	358(3)
46	AT2G28190.1	CSD2, CZSOD2 | copper/zinc superoxide dis	1350(5)			
47	AT4G26840.1	SUM1, SUMO 1, SUMO1, ATSUMO1 | small u	1312(7)	342(3)	138(6)	164(1)
48	AT5G20290.1	| Ribosomal protein S8e family protein | ch	1296(10)	204(3)	53(2)	
49	AT4G20260.6	PCAP1 | plasma-membrane associated cation-	1274(17)		71(7)	

50	AT2G19730.2	| Ribosomal L28e protein family | chr2:851	1261(7)			
51	AT2G19730.3	| Ribosomal L28e protein family | chr2:851	1261(7)			
52	AT2G19730.1	| Ribosomal L28e protein family | chr2:851	1261(7)			
53	AT4G32610.1	| copper ion binding | chr4:15728376-1572	1151(11)	206(4)	70(2)	
54	AT3G56910.1	PSRP5 | plastid-specific 50S ribosomal prot	1149(9)		635(4)	
55	AT2G17560.3	HMGB4, NFD4, NFD04 | high mobility group	742(15)	1138(14)	98(6)	
56	AT2G17560.2	HMGB4, NFD4, NFD04 | high mobility group	742(15)	1138(14)	98(6)	
57	AT2G17560.1	HMGB4, NFD4, NFD04 | high mobility group	742(15)	1138(14)	98(6)	
58	AT5G07530.1	GRP17, ATGRP17, ATGRP-7 | glycine rich pr	1136(18)			
59	AT5G22880.1	H2B, HTB2 | histone B2 | chr5:7652130-76	1074(15)	526(6)	50(4)	
60	AT3G52590.1	UBQ1, EMB2167, ERD16, HAP4 | ubiquitin ex	1039(10)	1045(4)		163(1)
61	AT2G36170.1	| Ubiquitin supergroup;Ribosomal protein L	1039(10)	1045(4)		163(1)
62	AT2G03680.2	SPR1, SKU6 | spiral1 | chr2:1121398-1121	1017(5)	979(5)	55(4)	
63	AT2G03680.1	SPR1, SKU6 | spiral1 | chr2:1121398-1121	1017(5)	979(5)	55(4)	
64	AT2G36160.1	| Ribosomal protein S11 family protein | c	1009(12)	525(7)	154(3)	
65	AT2G28720.1	| Histone superfamily protein | chr2:12327	1009(13)	526(7)	88(4)	
66	AT1G20450.2	LTI29, LTI45, ERD10 | Dehydrin family prot	1007(8)	82(4)	231(6)	
67	AT1G20450.1	LTI29, LTI45, ERD10 | Dehydrin family prot	1007(8)	82(4)	231(5)	
68	AT3G11510.1	| Ribosomal protein S11 family protein | ch	996(12)	525(7)	154(3)	
69	AT3G04920.1	| Ribosomal protein S24e family protein |	996(10)	625(9)	105(5)	
70	AT2G24090.1	| Ribosomal protein L35 | chr2:10242038-1	407(2)	990(3)		73(2)
71	AT1G31340.1	RUB1, NEDD8, ATRUB1 | related to ubiquiti	990(11)	679(2)		163(1)
72	AT1G22840.1	CYTC-1, ATCYTC-A | CYTOCHROME C-1 | ch	393(6)	970(4)	105(2)	308(2)
73	AT4G20260.5	PCAP1 | plasma-membrane associated cation-	965(14)		169(9)	
74	AT5G59910.1	HTB4 | Histone superfamily protein | chr5	964(18)	526(5)	88(6)	
75	AT3G45980.1	H2B, HTB9 | Histone superfamily protein |	947(17)	526(5)	101(6)	
76	AT2G35635.1	UBQ7, RUB2 | ubiquitin 7 | chr2:14981044	942(11)	679(2)		163(1)
77	AT3G46030.1	HTB11 | Histone superfamily protein | chr3	932(17)	526(5)	101(6)	
78	AT1G07790.1	HTB1 | Histone superfamily protein | chr1	928(21)	526(7)	79(2)	
79	AT5G15780.1	| Pollen Ole e 1 allergen and extensin famil	95(6)	920(4)		
80	AT1G74060.1	| Ribosomal protein L6 family protein | chr	916(18)		78(8)	
81	AT1G74050.1	| Ribosomal protein L6 family protein | chr	916(18)		78(8)	
82	AT5G07530.2	GRP17, ATGRP17, ATGRP-7 | glycine rich pr	905(15)			
83	AT1G65350.1	UBQ13 | ubiquitin 13 | chr1:24272518-242	891(10)	679(2)		163(1)
84	AT5G56670.1	| Ribosomal protein S30 family protein | c	466(6)	865(4)		71(1)
85	AT4G29390.1	| Ribosomal protein S30 family protein | c	466(6)	865(4)		71(1)
86	AT2G19750.1	| Ribosomal protein S30 family protein | c	466(6)	865(4)		71(1)
87	AT2G18050.1	HIS1-3 | histone H1-3 | chr2:7846095-784	862(16)	405(7)	74(10)	64(4)
88	AT5G16470.1	| zinc finger (C2H2 type) family protein |	849(8)	730(6)	679(5)	55(1)
89	AT1G22450.1	COX6B, ATCOX6B2 | cytochrome C oxidase 6	849(8)		84(6)	
90	AT4G35570.1	HMGB5, NFD5, HMGD, NFD05 | high mobility	846(16)	459(6)	364(5)	330(2)
91	AT5G37780.1	CAM1, TCH1, ACAM-1 | calmodulin 1 | chr5	846(9)	127(2)		
92	AT1G66410.1	CAM4, ACAM-4 | calmodulin 4 | chr1:24774	846(9)	127(2)		
93	AT2G47110.2	UBQ6 | ubiquitin 6 | chr2:19344701-19345	845(10)	719(3)		217(2)
94	AT2G47110.1	UBQ6 | ubiquitin 6 | chr2:19344701-19345	845(10)	719(3)		217(2)
95	AT3G62250.1	UBQ5 | ubiquitin 5 | chr3:23037138-23037	845(10)	719(3)		167(2)
96	AT1G23410.1	| Ribosomal protein S27a / Ubiquitin family	845(10)	679(2)		217(2)
97	AT3G43810.1	CAM7 | calmodulin 7 | chr3:15664619-1566	840(9)	127(2)		
98	AT5G21274.1	CAM6, ACAM-6 | calmodulin 6 | chr5:72147	840(9)	127(2)		
99	AT2G41110.1	CAM2, ATCAL5 | calmodulin 2 | chr2:17140	840(9)	127(2)		
100	AT2G27030.1	CAM5, ACAM-2 | calmodulin 5 | chr2:11532	840(9)	127(2)		
101	AT2G27030.3	CAM5 | calmodulin 5 | chr2:11532069-1153	840(9)	127(2)		
102	AT3G56800.1	CAM3, acam-3 | calmodulin 3 | chr3:210349	840(9)	127(2)		
103	AT4G05320.1	UBQ10 | polyubiquitin 10 | chr4:2718559-	834(9)	679(2)		163(1)

104	AT4G05320.3	UBQ10 | polyubiquitin 10 | chr4:2718787-	834(9)	679(2)		163(1)
105	AT4G05320.2	UBQ10 | polyubiquitin 10 | chr4:2718559-	834(9)	679(2)		163(1)
106	AT4G05320.5	UBQ10 | polyubiquitin 10 | chr4:2718559-	834(9)	679(2)		163(1)
107	AT4G05320.4	UBQ10 | polyubiquitin 10 | chr4:2718559-	834(9)	679(2)		163(1)
108	AT4G05320.6	UBQ10 | polyubiquitin 10 | chr4:2718559-	834(9)	679(2)		163(1)
109	AT4G02890.2	UBQ14 | Ubiquitin family protein | chr4:12	834(9)	679(2)		163(1)
110	AT4G02890.1	UBQ14 | Ubiquitin family protein | chr4:12	834(9)	679(2)		163(1)
111	AT4G02890.4	UBQ14 | Ubiquitin family protein | chr4:12	834(9)	679(2)		163(1)
112	AT4G02890.3	UBQ14 | Ubiquitin family protein | chr4:12	834(9)	679(2)		163(1)
113	AT1G55060.1	UBQ12 | ubiquitin 12 | chr1:20549533-205	834(9)	679(2)		
114	AT5G03240.3	UBQ3 | polyubiquitin 3 | chr5:771976-7728	834(9)	679(2)		163(1)
115	AT5G03240.2	UBQ3 | polyubiquitin 3 | chr5:771976-7728	834(9)	679(2)		163(1)
116	AT5G03240.1	UBQ3 | polyubiquitin 3 | chr5:771976-7728	834(9)	679(2)		163(1)
117	AT5G37640.1	UBQ9 | ubiquitin 9 | chr5:14952782-149537	834(10)	679(2)		
118	AT4G05050.1	UBQ11 | ubiquitin 11 | chr4:2588271-25889	834(9)	679(2)		163(1)
119	AT4G05050.3	UBQ11 | ubiquitin 11 | chr4:2588271-25889	834(9)	679(2)		163(1)
120	AT4G05050.2	UBQ11 | ubiquitin 11 | chr4:2588271-25889	834(9)	679(2)		163(1)
121	AT4G05050.4	UBQ11 | ubiquitin 11 | chr4:2588271-25889	834(9)	679(2)		163(1)
122	AT5G20620.1	UBQ4 | ubiquitin 4 | chr5:6973315-6974463	834(9)	679(2)		163(1)
123	AT2G27730.1	| copper ion binding | chr2:11820056-1182	821(4)	261(7)	62(7)	
124	AT5G37780.3	CAM1, TCH1, ACAM-1 | calmodulin 1 | chr5	821(8)	127(2)		
125	AT5G37780.2	CAM1, TCH1, ACAM-1 | calmodulin 1 | chr5	821(8)	127(2)		
126	AT1G66410.2	CAM4 | calmodulin 4 | chr1:24774431-2477	821(8)	127(2)		
127	AT3G09790.1	UBQ8 | ubiquitin 8 | chr3:3004111-3006006	820(9)	679(2)		
128	AT3G47070.1	| LOCATED IN: thylakoid, chloroplast thylako	819(8)	140(8)	141(2)	
129	AT2G41110.2	CAM2 | calmodulin 2 | chr2:17140379-1714	815(8)	127(2)		
130	AT5G07030.1	| Eukaryotic aspartyl protease family prote	795(16)	239(10)	88(7)	
131	AT3G52580.1	| Ribosomal protein S11 family protein | c	783(11)	525(7)	154(5)	
132	AT5G23900.1	| Ribosomal protein L13e family protein | c	779(10)	53(2)	53(3)	
133	AT1G12080.2	| Vacuolar calcium-binding protein-related	772(6)			
134	AT2G45820.1	| Remorin family protein | chr2:18863147-1	752(4)			
135	AT1G08580.1	| unknown protein; Has 39 Blast hits to 39 pr	738(4)	55(1)		
136	AT5G18100.2	CSD3 | copper/zinc superoxide dismutase 3	732(3)	263(1)	86(2)	
137	AT5G18100.1	CSD3 | copper/zinc superoxide dismutase 3	732(3)	263(1)		
138	AT5G64130.1	| cAMP-regulated phosphoprotein 19-related	731(4)	291(6)	57(2)	
139	AT5G64130.3	| cAMP-regulated phosphoprotein 19-related	731(4)	291(6)	57(2)	
140	AT3G12390.1	| Nascent polypeptide-associated complex (N	715(10)			
141	AT3G44100.1	| MD-2-related lipid recognition domain-con	710(11)	322(5)		
142	AT2G27030.2	CAM5 | calmodulin 5 | chr2:11532719-1153	706(5)	127(2)		
143	AT3G15790.1	MBD11, ATMBD11 | methyl-CPG-binding dom	703(9)	72(4)	95(5)	
144	AT1G18540.1	| Ribosomal protein L6 family protein | chr	689(17)			
145	AT1G03600.1	PSB27 | photosystem II family protein | ch	668(4)	330(3)		
146	AT4G28750.1	PSAE-1 | Photosystem I reaction centre subu	652(9)	374(9)	392(5)	
147	AT1G47200.1	WPP2 | WPP domain protein 2 | chr1:17298	651(5)	60(2)		
148	AT3G49010.5	ATBBC1, BBC1, RSU2 | breast basic conserv	649(9)	136(2)	58(3)	358(3)
149	AT2G18050.2	HIS1-3 | histone H1-3 | chr2:7846254-784	643(12)	333(3)	51(5)	64(3)
150	AT2G30410.1	KIS, TFCA | tubulin folding cofactor A (KI	638(12)	378(7)	489(10)	
151	AT2G30410.2	KIS | tubulin folding cofactor A (KIESEL)	638(12)	378(7)	489(10)	
152	AT2G27710.2	| 60S acidic ribosomal protein family | ch	617(11)			
153	AT2G27710.1	| 60S acidic ribosomal protein family | ch	617(11)			
154	AT2G27710.3	| 60S acidic ribosomal protein family | ch	617(11)			
155	AT1G22840.2	CYTC-1, ATCYTC-A | CYTOCHROME C-1 | ch	393(4)	601(3)	105(2)	
156	AT2G27720.1	| 60S acidic ribosomal protein family | ch	596(14)		101(4)	
157	AT5G13850.1	NACA3 | nascent polypeptide-associated comp	595(11)			

158	AT3G09500.1	| Ribosomal L29 family protein | chr3:2917	590(15)	176(6)		157(3)
159	AT1G09310.1	| Protein of unknown function, DUF538 | c	571(10)	88(4)		
160	AT2G03440.1	NRP1, ATNRP1 | nodulin-related protein 1	547(10)	282(6)	57(4)	
161	AT3G09480.1	| Histone superfamily protein | chr3:29148	147(8)	526(8)		
162	AT5G02570.1	| Histone superfamily protein | chr5:57674	348(11)	526(6)		
163	AT2G37470.1	| Histone superfamily protein | chr2:15736	366(13)	526(6)		
164	AT2G27710.4	| 60S acidic ribosomal protein family | ch	511(10)			
165	AT4G18100.1	| Ribosomal protein L32e | chr4:10035715-	503(5)			
166	AT1G02780.1	emb2386 | Ribosomal protein L19e family pr	473(6)	187(8)		
167	AT3G49910.1	| Translation protein SH3-like family prote	465(11)	333(5)		
168	AT5G55160.1	SUM2, SUMO 2, SUMO2, ATSUMO2 | small u	457(7)	52(1)		
169	AT1G69510.1	| cAMP-regulated phosphoprotein 19-related	430(8)	74(4)	73(2)	63(1)
170	AT1G69510.3	| cAMP-regulated phosphoprotein 19-related	430(8)	74(4)	73(2)	63(1)
171	AT1G69510.2	| cAMP-regulated phosphoprotein 19-related	430(8)	74(4)	73(2)	63(1)
172	AT3G55170.1	| Ribosomal L29 family protein | chr3:2045	430(11)	176(6)		157(2)
173	AT3G55170.2	| Ribosomal L29 family protein | chr3:2045	430(11)	176(6)		157(2)
174	AT4G02770.1	PSAD-1 | photosystem I subunit D-1 | chr4	430(6)			
175	AT4G31880.1	| LOCATED IN: cytosol, chloroplast; EXPRESS	426(19)			
176	AT4G31880.2	| LOCATED IN: cytosol; EXPRESSED IN: 24 p	426(19)			
177	AT5G51720.1	| 2 iron, 2 sulfur cluster binding | chr5:2	425(6)	171(4)		
178	AT1G19990.1	| unknown protein; FUNCTIONS IN: molecular_	420(16)			
179	AT1G03130.1	PSAD-2 | photosystem I subunit D-2 | chr1	419(5)			
180	AT4G31700.1	RPS6, RPS6A | ribosomal protein S6 | chr4	417(7)	188(3)		
181	AT5G18540.1	| unknown protein; Has 2026 Blast hits to 792	415(5)	67(7)		
182	AT4G16500.1	| Cystatin/monellin superfamily protein | c	410(8)	70(1)	58(2)	
183	AT1G69230.2	SP1L2 | SPIRAL1-like2 | chr1:26026543-26	410(1)	225(1)	56(1)	
184	AT1G69230.1	SP1L2 | SPIRAL1-like2 | chr1:26026543-26	410(1)	225(1)	56(1)	
185	AT2G39460.2	ATRPL23A, RPL23A, RPL23AA | ribosomal pr	406(7)			
186	AT2G39460.1	ATRPL23A, RPL23A, RPL23AA | ribosomal pr	406(7)			
187	AT3G55280.1	RPL23AB | ribosomal protein L23AB | chr3	406(7)			
188	AT3G55280.2	RPL23AB | ribosomal protein L23AB | chr3	406(7)			
189	AT3G55280.3	RPL23AB | ribosomal protein L23AB | chr3	406(8)			
190	AT5G18540.2	| unknown protein; Has 1784 Blast hits to 634	391(4)	67(7)		
191	AT5G55730.1	FLA1 | FASCICLIN-like arabinogalactan 1 |	389(8)			
192	AT5G55730.2	FLA1 | FASCICLIN-like arabinogalactan 1 |	389(8)			
193	AT2G38540.1	LP1, LTP1, ATLTP1 | lipid transfer protein	169(4)	382(7)		
194	AT1G04270.2	RPS15 | cytosolic ribosomal protein S15 |	379(5)	141(8)		
195	AT1G04270.1	RPS15 | cytosolic ribosomal protein S15 |	379(5)	141(4)		
196	AT5G46430.2	| Ribosomal protein L32e | chr5:18833429-	373(4)			
197	AT5G46430.1	| Ribosomal protein L32e | chr5:18833429-	373(4)			
198	AT5G15970.1	KIN2, COR6.6 | stress-responsive protein (KIN	372(6)			61(3)
199	AT5G02770.1	| unknown protein; Has 469 Blast hits to 336	372(4)			
200	AT5G60030.1	| unknown protein; BEST Arabidopsis thaliana p	372(7)			
201	AT2G45470.1	FLA8, AGP8 | FASCICLIN-like arabinogalacta	367(12)		67(4)	
202	AT2G42680.1	MBF1A, ATMBF1A | multiprotein bridging fa	365(6)	132(5)		
203	AT5G09510.1	| Ribosomal protein S19 family protein | ch	348(4)	141(3)		
204	AT2G27720.3	| 60S acidic ribosomal protein family | ch	346(12)		101(4)	
205	AT2G27720.2	| 60S acidic ribosomal protein family | ch	346(12)		97(2)	
206	AT4G21280.1	PSBQ, PSBQA, PSBQ-1 | photosystem II sub	345(12)	59(6)	67(2)	
207	AT3G51600.1	LTP5 | lipid transfer protein 5 | chr3:1913	78(3)	342(6)		
208	AT2G21580.2	| Ribosomal protein S25 family protein | c	341(10)	316(4)		
209	AT2G21580.1	| Ribosomal protein S25 family protein | c	341(10)	316(4)		
210	AT5G66570.1	PSBO-1, OEE1, OEE33, OE33, PSBO1, MSP-	336(8)			
211	AT3G50820.1	PSBO2, PSBO-2, OEC33 | photosystem II sub	336(8)			

212	AT3G53650.1	| Histone superfamily protein | chr3:19889	325(9)	128(4)		
213	AT5G28060.1	| Ribosomal protein S24e family protein |	260(6)	323(4)	96(5)	
214	AT3G23390.1	| Zinc-binding ribosomal protein family pro	322(4)		57(3)	
215	AT4G14320.1	| Zinc-binding ribosomal protein family pro	322(4)		57(3)	
216	AT2G47400.1	CP12-1, CP12 | CP12 domain-containing pro	322(3)			
217	AT3G49470.1	NACA2 | nascent polypeptide-associated comp	318(6)	85(1)		
218	AT4G21280.2	PSBQ, PSBQA, PSBQ-1 | photosystem II sub	316(11)	59(4)	54(1)	
219	AT4G34555.1	| Ribosomal protein S25 family protein | c	181(10)	316(4)		
220	AT4G14320.2	| Zinc-binding ribosomal protein family pro	314(3)		57(3)	
221	AT1G22780.1	PFL, RPS18A, PFL1 | Ribosomal protein S13	308(5)	151(2)		
222	AT4G09800.1	RPS18C | S18 ribosomal protein | chr4:617	308(5)	151(2)		
223	AT1G34030.1	| Ribosomal protein S13/S18 family | chr1:	308(5)	151(2)		
224	AT3G20390.1	| endoribonuclease L-PSP family protein |	305(9)		129(5)	
225	AT5G55160.2	SUM2, SUMO 2, SUMO2, ATSUMO2 | small u	300(5)	52(1)		
226	AT5G11420.1	| Protein of unknown function, DUF642 | c	295(9)			
227	AT1G26880.1	| Ribosomal protein L34e superfamily protei	136(6)	291(19)		
228	AT5G42980.1	ATTRX3, ATH3, ATTRXH3, TRXH3, TRX3 | thi	290(6)			
229	AT1G31812.1	ACBP6, ACBP | acyl-CoA-binding protein 6	284(5)		90(2)	
230	AT4G39200.2	| Ribosomal protein S25 family protein | c	282(11)	142(4)		
231	AT4G39200.1	| Ribosomal protein S25 family protein | c	282(11)	142(4)		
232	AT3G58680.1	MBF1B, ATMBF1B | multiprotein bridging fa	276(5)	150(5)		
233	AT4G27090.1	| Ribosomal protein L14 | chr4:13594104-1	273(7)		67(1)	
234	AT1G69620.1	RPL34 | ribosomal protein L34 | chr1:2618	87(6)	262(7)		
235	AT1G22530.1	PATL2 | PATELLIN 2 | chr1:7955773-79583	256(7)			
236	AT3G02790.1	| zinc finger (C2H2 type) family protein |	54(1)	255(2)		
237	AT3G04120.1	GAPC, GAPC-1, GAPC1 | glyceraldehyde-3-ph	253(8)			
238	AT1G13440.1	GAPC-2, GAPC2 | glyceraldehyde-3-phosphat	253(8)			
239	AT1G13440.2	GAPC-2, GAPC2 | glyceraldehyde-3-phosphat	253(8)			
240	AT3G02180.2	SP1L3 | SPIRAL1-like3 | chr3:404989-4054	134(2)	251(4)	57(2)	94(3)
241	AT3G02180.1	SP1L3 | SPIRAL1-like3 | chr3:404989-4054	134(2)	251(4)	57(2)	94(3)
242	AT3G20670.1	HTA13 | histone H2A 13 | chr3:7229472-72	250(9)	99(3)		
243	AT1G33590.1	| Leucine-rich repeat (LRR) family protein	250(10)			
244	AT1G01490.2	| Heavy metal transport/detoxification super	250(9)			
245	AT1G01490.1	| Heavy metal transport/detoxification super	250(9)			
246	AT4G37300.1	MEE59 | maternal effect embryo arrest 59 |	243(3)	155(3)		
247	AT1G12080.1	| Vacuolar calcium-binding protein-related	243(3)			
248	AT5G10360.2	EMB3010 | Ribosomal protein S6e | chr5:3	238(6)	169(6)		
249	AT5G10360.1	EMB3010, RPS6B | Ribosomal protein S6e |	238(6)	169(6)		
250	AT5G62350.1	| Plant invertase/pectin methylesterase inhi	198(6)	237(5)		
251	AT4G10480.2	| Nascent polypeptide-associated complex (NA	236(4)			
252	AT4G10480.1	| Nascent polypeptide-associated complex (NA	236(4)			
253	AT4G31700.2	RPS6, RPS6A | ribosomal protein S6 | chr4	234(4)	188(3)		
254	AT3G15190.1	| chloroplast 30S ribosomal protein S20, pu	232(7)			
255	AT3G54400.1	| Eukaryotic aspartyl protease family prote	229(7)	107(8)	115(4)	78(2)
256	AT3G61260.1	| Remorin family protein | chr3:22675403-2	229(4)	72(2)		
257	AT4G27230.2	HTA2 | histone H2A 2 | chr4:13637515-136	228(7)	122(4)		66(4)
258	AT4G27230.1	HTA2 | histone H2A 2 | chr4:13637515-136	228(7)	122(4)		66(4)
259	AT3G56240.1	CCH | copper chaperone | chr3:20863460-2	228(6)			
260	AT3G28830.1	| Protein of unknown function (DUF1216) |	225(6)			
261	AT1G26880.2	| Ribosomal protein L34e superfamily protei	107(5)	224(7)		
262	AT1G56660.1	| unknown protein; Has 665200 Blast hits to 20	222(8)			
263	AT1G51060.1	HTA10 | histone H2A 10 | chr1:18926948-1	221(6)	154(4)		105(5)
264	AT5G54640.1	HTA1, RAT5, ATHTA1 | Histone superfamily	218(5)	99(3)		63(4)
265	AT5G59320.1	LTP3 | lipid transfer protein 3 | chr5:239	217(1)	58(2)		

266	AT3G16850.1	| Pectin lyase-like superfamily protein | c	213(7)			
267	AT4G10040.1	CYTC-2 | cytochrome c-2 | chr4:6277083-6	189(4)	207(2)		
268	AT5G26000.2	TGG1, BGLU38 | thioglucoside glucohydrolas	207(5)	58(2)		
269	AT5G26000.1	TGG1, BGLU38 | thioglucoside glucohydrolas	207(5)	58(2)		
270	AT2G20450.1	| Ribosomal protein L14 | chr2:8813923-88	206(5)		67(1)	
271	AT4G39260.1	CCR1, ATGRP8, GR-RBP8, GRP8 | cold, circa	171(4)	199(4)		
272	AT4G39260.2	CCR1, ATGRP8, GR-RBP8, GRP8 | cold, circa	171(4)	199(4)		
273	AT4G39260.3	CCR1, ATGRP8, GR-RBP8, GRP8 | cold, circa	171(4)	199(4)		
274	AT1G47128.1	RD21, RD21A | Granulin repeat cysteine prot	197(2)		59(1)	
275	AT2G21660.2	ATGRP7, CCR2 | cold, circadian rhythm, and	187(3)	194(2)		
276	AT2G21660.1	ATGRP7, CCR2, GR-RBP7, GRP7 | cold, circad	187(3)	194(2)		
277	AT5G02610.1	| Ribosomal L29 family protein | chr5:5876	194(12)	131(3)		100(2)
278	AT5G02610.2	| Ribosomal L29 family protein | chr5:5876	194(12)	131(3)		100(2)
279	AT1G72150.1	PATL1 | PATELLIN 1 | chr1:27148558-2715	192(12)			
280	AT1G17880.1	BTF3, ATBTF3 | basic transcription factor 3	188(8)			
281	AT4G39260.4	CCR1, ATGRP8, GR-RBP8, GRP8 | cold, circa	152(3)	186(2)		
282	AT2G39390.1	| Ribosomal L29 family protein | chr2:1645	186(11)	131(4)		100(2)
283	AT1G03870.1	FLA9 | FASCICLIN-like arabinoogalactan 9	186(6)			
284	AT5G02560.1	HTA12 | histone H2A 12 | chr5:575437-576	183(6)	170(15)	123(5)	
285	AT2G38140.1	PSRP4 | plastid-specific ribosomal protein	175(5)	62(2)		
286	AT3G28900.1	| Ribosomal protein L34e superfamily protei	66(4)	174(4)		
287	AT5G60390.1	| GTP binding Elongation factor Tu family p	174(7)	77(7)		
288	AT5G60390.3	| GTP binding Elongation factor Tu family p	174(7)	77(7)		
289	AT1G07930.1	| GTP binding Elongation factor Tu family p	174(7)	77(7)		
290	AT1G07920.1	| GTP binding Elongation factor Tu family p	174(7)	77(7)		
291	AT1G07940.1	| GTP binding Elongation factor Tu family p	174(7)	77(7)		
292	AT1G07940.2	| GTP binding Elongation factor Tu family p	174(7)	77(7)		
293	AT4G12730.1	FLA2 | FASCICLIN-like arabinogalactan 2 |	173(6)			
294	AT2G16360.1	| Ribosomal protein S25 family protein | ch	172(6)	126(2)		
295	AT3G12145.1	FLR1, FLOR1 | Leucine-rich repeat (LRR) fam	171(4)	83(1)		
296	AT5G02560.2	HTA12 | histone H2A 12 | chr5:575437-576	102(5)	170(15)	123(5)	
297	AT1G07930.2	| GTP binding Elongation factor Tu family p	170(6)	79(4)		
298	AT2G18020.1	EMB2296 | Ribosomal protein L2 family | c	169(4)	94(2)		
299	AT3G20820.1	| Leucine-rich repeat (LRR) family protein	145(7)	168(10)	52(2)	
300	AT3G05560.2	| Ribosomal L22e protein family | chr3:161	130(7)	168(1)		
301	AT3G05560.1	| Ribosomal L22e protein family | chr3:161	130(7)	168(1)		
302	AT3G05560.3	| Ribosomal L22e protein family | chr3:161	130(7)	168(1)		
303	AT5G27770.1	| Ribosomal L22e protein family | chr5:983	143(6)	168(1)		
304	AT5G25460.1	| Protein of unknown function, DUF642 | c	168(8)			
305	AT1G71695.1	| Peroxidase superfamily protein | chr1:26	53(1)	167(4)		
306	AT2G33830.2	| Dormancy/auxin associated family protein	167(2)	56(3)		
307	AT1G21500.1	| unknown protein; Has 29 Blast hits to 29 pr	144(3)	166(2)	73(4)	
308	AT5G47210.1	| Hyaluronan / mRNA binding family | chr5	165(4)			
309	AT3G53730.1	| Histone superfamily protein | chr3:19912	163(3)			
310	AT5G59690.1	| Histone superfamily protein | chr5:24051	163(3)			
311	AT1G07660.1	| Histone superfamily protein | chr1:23692	163(3)			
312	AT3G45930.1	| Histone superfamily protein | chr3:16883	163(3)			
313	AT2G28740.1	HIS4 | histone H4 | chr2:12329643-123299	163(3)			
314	AT3G46320.1	| Histone superfamily protein | chr3:17020	163(3)			
315	AT1G07820.2	| Histone superfamily protein | chr1:24214	163(3)			
316	AT1G07820.1	| Histone superfamily protein | chr1:24214	163(3)			
317	AT5G59970.1	| Histone superfamily protein | chr5:24146	163(3)			
318	AT5G47210.3	| Hyaluronan / mRNA binding family | chr5	161(3)			
319	AT1G16030.1	Hsp70b | heat shock protein 70B | chr1:55	160(4)			

320	AT5G02500.1	HSC70-1, HSP70-1, AT-HSC70-1, HSC70 | he	160(5)			
321	AT5G02500.2	HSC70-1, HSP70-1, AT-HSC70-1, HSC70 | he	160(5)			
322	AT3G12580.1	HSP70, ATHSP70 | heat shock protein 70 |	160(5)			
323	AT1G56410.1	ERD2, HSP70T-1 | heat shock protein 70 (Hs	160(4)			
324	AT3G09440.2	| Heat shock protein 70 (Hsp 70) family pro	160(5)			
325	AT3G09440.1	| Heat shock protein 70 (Hsp 70) family pro	160(5)			
326	AT5G02490.1	| Heat shock protein 70 (Hsp 70) family pro	160(5)			
327	AT1G71950.1	| Proteinase inhibitor, propeptide | chr1:2	160(3)			
328	AT3G07470.1	| Protein of unknown function, DUF538 | ch	159(4)	58(1)		59(1)
329	AT5G59870.1	HTA6 | histone H2A 6 | chr5:24115605-241	54(4)	158(6)		
330	AT3G57930.2	| unknown protein; BEST Arabidopsis thaliana p	158(5)	119(3)		
331	AT3G57930.1	| unknown protein; BEST Arabidopsis thaliana p	158(5)	119(3)		
332	AT3G14210.1	ESM1 | epithiospecifier modifier 1 | chr3:	156(7)			
333	AT5G27850.1	| Ribosomal protein L18e/L15 superfamily p	155(7)	131(5)		56(1)
334	AT5G20740.1	| Plant invertase/pectin methylesterase inhib	154(3)			
335	AT4G03520.1	ATHM2 | Thioredoxin superfamily protein |	154(4)			
336	AT4G02230.1	| Ribosomal protein L19e family protein | c	141(5)	151(4)		
337	AT2G30860.1	ATGSTF9, GLUTTR, ATGSTF7, GSTF9 | glutat	150(6)			
338	AT1G73230.1	| Nascent polypeptide-associated complex N	149(9)		59(6)	
339	AT5G47550.1	| Cystatin/monellin superfamily protein | c	62(3)	146(1)		
340	AT4G05180.1	PSBQ, PSBQ-2, PSII-Q | photosystem II sub	146(8)	55(3)		
341	AT4G13560.1	UNE15 | Late embryogenesis abundant protein	145(6)	57(2)		
342	AT5G17920.1	ATCIMS, ATMETS, ATMS1 | Cobalamin-indepe	144(3)			
343	AT5G17920.2	ATCIMS | Cobalamin-independent synthase fa	144(3)			
344	AT4G29410.2	| Ribosomal L28e protein family | chr4:144	144(6)			
345	AT4G29410.1	| Ribosomal L28e protein family | chr4:144	144(6)			
346	AT5G39740.2	RPL5B | ribosomal protein L5 B | chr5:159	109(6)			143(1)
347	AT5G39740.1	OLI7, RPL5B | ribosomal protein L5 B | ch	109(6)			143(1)
348	AT3G25520.1	ATL5, PGY3, OLI5, RPL5A | ribosomal protei	109(10)			143(1)
349	AT5G20630.1	GLP3, GLP3A, GLP3B, ATGER3, GER3 | germi	140(2)	116(3)		
350	AT5G27670.1	HTA7 | histone H2A 7 | chr5:9792807-9793	97(4)	138(7)		
351	AT1G29670.1	| GDSL-like Lipase/Acylhydrolase superfami	135(4)	118(6)		
352	AT3G05590.1	RPL18 | ribosomal protein L18 | chr3:1621	129(5)	131(4)		56(1)
353	AT5G42020.1	BIP, BIP2 | Heat shock protein 70 (Hsp 70)	131(3)			
354	AT5G42020.2	BIP | Heat shock protein 70 (Hsp 70) family	131(3)			
355	AT4G03520.2	ATHM2 | Thioredoxin superfamily protein |	127(3)			
356	AT1G55265.1	| Protein of unknown function, DUF538 | c	126(5)			
357	AT4G15802.1	HSBP, AtHSBP | heat shock factor binding p	125(5)	81(3)		
358	AT2G36620.1	RPL24A | ribosomal protein L24 | chr2:153	123(3)			
359	AT3G53020.1	STV1, RPL24B, RPL24 | Ribosomal protein L2	123(4)			
360	AT5G58070.1	ATTIL, TIL | temperature-induced lipocalin	122(5)	66(2)		
361	AT1G75040.1	PR5, PR-5 | pathogenesis-related gene 5 |	67(3)	121(5)		
362	AT1G17860.1	| Kunitz family trypsin and protease inhibit	118(9)	71(3)		
363	AT1G08880.1	HTA5, H2AXA, G-H2AX, GAMMA-H2AX | Hist	117(5)	103(5)	79(1)	62(5)
364	AT1G54690.1	HTA3, H2AXB, G-H2AX, GAMMA-H2AX | gam	117(5)	103(5)	59(1)	62(5)
365	AT1G24020.1	MLP423 | MLP-like protein 423 | chr1:8500	117(4)			
366	AT1G24020.2	MLP423 | MLP-like protein 423 | chr1:8500	117(4)			
367	AT2G30860.2	ATGSTF9, GLUTTR, ATGSTF7, GSTF9 | glutat	116(5)			
368	AT2G02930.1	ATGSTF3, GST16, GSTF3 | glutathione S-tra	66(4)	109(2)		
369	AT4G02520.1	ATGSTF2, ATPM24.1, ATPM24, GST2, GSTF2	105(7)	109(2)		
370	AT5G09810.1	ACT7 | actin 7 | chr5:3052809-3054220 FO	98(6)	109(7)		
371	AT1G68560.1	ATXYL1, XYL1, TRG1 | alpha-xylosidase 1 |	109(4)			
372	AT3G62820.1	| Plant invertase/pectin methylesterase inhi	109(3)			
373	AT5G38420.1	| Ribulose bisphosphate carboxylase (small c	106(6)			

374	AT5G38410.3	| Ribulose bisphosphate carboxylase (small c	106(6)			
375	AT5G38410.1	| Ribulose bisphosphate carboxylase (small c	106(6)			
376	AT5G38410.2	| Ribulose bisphosphate carboxylase (small c	106(6)			
377	AT5G24165.1	| unknown protein; FUNCTIONS IN: molecular_	96(3)	105(5)	68(2)	
378	AT5G40370.1	| Glutaredoxin family protein | chr5:16147	105(3)			
379	AT5G40370.2	| Glutaredoxin family protein | chr5:16147	105(3)			
380	AT1G67090.1	RBCS1A | ribulose bisphosphate carboxylase	105(4)			
381	AT5G03170.1	FLA11, ATFLA11 | FASCICLIN-like arabinogal	105(3)			
382	AT4G00430.1	TMP-C, PIP1;4, PIP1E | plasma membrane int	71(2)	104(2)		
383	AT2G45960.1	PIP1B, TMP-A, ATHH2, PIP1;2 | plasma memb	90(3)	104(2)		
384	AT2G45960.3	PIP1B, TMP-A, ATHH2, PIP1;2 | plasma memb	90(3)	104(2)		
385	AT2G45960.2	PIP1B, TMP-A, ATHH2, PIP1;2 | plasma memb	90(3)	104(2)		
386	AT1G01620.1	PIP1C, TMP-B, PIP1;3 | plasma membrane int	71(2)	104(2)		
387	AT3G61430.2	PIP1A, ATPIP1, PIP1, PIP1;1 | plasma membr	90(3)	104(2)		
388	AT3G61430.1	PIP1A, ATPIP1, PIP1, PIP1;1 | plasma membr	90(3)	104(2)		
389	AT1G67090.2	RBCS1A | ribulose bisphosphate carboxylase	103(3)			
390	AT3G63160.1	| FUNCTIONS IN: molecular_function unknown	102(3)		73(5)	
391	AT1G03680.1	ATHM1, TRX-M1, ATM1, THM1 | thioredoxin	100(6)			
392	AT3G32980.1	| Peroxidase superfamily protein | chr3:13	76(6)	99(5)		
393	AT5G37720.1	ALY4 | ALWAYS EARLY 4 | chr5:14981805-1	99(2)	72(2)		
394	AT5G37720.2	ALY4 | ALWAYS EARLY 4 | chr5:14981805-1	99(2)	72(2)		
395	AT3G52040.1	| unknown protein; Has 37 Blast hits to 37 pr	99(3)			58(1)
396	AT5G44130.1	FLA13 | FASCICLIN-like arabinogalactan pro	98(6)			
397	AT3G49110.1	PRX33, PRXCA, ATPRX33, ATPCA | peroxidas	97(6)	79(5)		
398	AT3G14310.1	ATPME3, PME3 | pectin methylesterase 3 |	97(6)			
399	AT2G04780.1	FLA7 | FASCICLIN-like arabinoogalactan 7	96(3)	64(1)		
400	AT2G04780.2	FLA7 | FASCICLIN-like arabinoogalactan 7	96(3)	64(1)		
401	AT4G13340.1	| Leucine-rich repeat (LRR) family protein	96(4)			
402	AT2G24940.1	AtMAPR2, MAPR2 | membrane-associated prog	96(3)			
403	AT5G09440.1	EXL4 | EXORDIUM like 4 | chr5:2938397-2	95(5)			
404	AT2G38810.3	HTA8 | histone H2A 8 | chr2:16219444-162	94(3)			
405	AT2G38810.2	HTA8 | histone H2A 8 | chr2:16219444-162	94(3)			
406	AT2G38810.1	HTA8 | histone H2A 8 | chr2:16219444-162	94(3)			
407	AT3G54560.1	HTA11 | histone H2A 11 | chr3:20196532-2	94(3)			
408	AT1G52740.1	HTA9 | histone H2A protein 9 | chr1:19645	94(3)			
409	AT4G21650.1	| Subtilase family protein | chr4:11501314	92(8)			
410	AT3G09680.1	| Ribosomal protein S12/S23 family protein	92(3)			
411	AT5G02960.1	| Ribosomal protein S12/S23 family protein	92(3)			
412	AT4G36130.1	| Ribosomal protein L2 family | chr4:17097	92(3)			
413	AT3G49120.1	ATPERX34, PERX34, PRXCB, ATPCB, PRX34	89(6)	90(5)		
414	ATCG00480.1	ATPB, PB | ATP synthase subunit beta | ch	90(9)	89(5)		
415	AT1G75190.1	| unknown protein; Has 7306 Blast hits to 385	89(3)			
416	AT1G75190.2	| unknown protein; FUNCTIONS IN: molecular_	89(3)			
417	AT5G63550.1	| DEK domain-containing chromatin associat	86(6)			
418	AT5G63550.2	| DEK domain-containing chromatin associat	86(6)			
419	AT1G07670.1	ATECA4, ECA4 | endomembrane-type CA-ATPa	86(4)			
420	AT4G23670.1	| Polyketide cyclase/dehydrase and lipid tra	86(3)			
421	AT5G17870.1	PSRP6 | plastid-specific 50S ribosomal pro	52(4)	85(1)		
422	AT3G24480.1	| Leucine-rich repeat (LRR) family protein	85(6)			
423	AT1G72290.1	| Kunitz family trypsin and protease inhibit	84(3)	71(2)		
424	AT4G11175.1	| Nucleic acid-binding, OB-fold-like protei	83(4)			
425	AT1G47960.1	C/VIF1, ATC/VIF1 | cell wall / vacuolar inhi	82(3)			
426	AT4G02620.1	| vacuolar ATPase subunit F family protein	79(3)			
427	AT5G15200.1	| Ribosomal protein S4 | chr5:4935124-493	77(7)			

428	AT3G60900.1	FLA10 | FASCICLIN-like arabinogalactan-pro	76(4)		67(4)	
429	AT3G47800.1	| Galactose mutarotase-like superfamily pro	76(4)			
430	AT1G17100.1	| SOUL heme-binding family protein | chr1	75(3)	58(1)		
431	AT1G67430.1	| Ribosomal protein L22p/L17e family prote	75(12)			
432	AT1G06680.1	PSBP-1, OEE2, PSII-P, OE23 | photosystem	73(4)			
433	AT5G43070.1	WPP1 | WPP domain protein 1 | chr5:17289	71(2)	64(1)		
434	AT2G47980.1	SCC3, ATSCC3 | sister-chromatid cohesion p	70(7)			
435	AT5G39850.1	| Ribosomal protein S4 | chr5:15950053-15	70(5)			
436	AT3G52930.1	| Aldolase superfamily protein | chr3:1962	70(3)			
437	AT4G27170.1	SESA4, AT2S4 | seed storage albumin 4 | c	53(2)	68(2)		
438	AT2G17360.1	| Ribosomal protein S4 (RPS4A) family prot	67(5)			
439	AT2G17360.2	| Ribosomal protein S4 (RPS4A) family prot	67(3)			
440	AT5G07090.1	| Ribosomal protein S4 (RPS4A) family prot	67(5)			
441	AT5G07090.2	| Ribosomal protein S4 (RPS4A) family prot	67(5)			
442	AT5G58420.1	| Ribosomal protein S4 (RPS4A) family prot	67(5)			
443	AT1G27400.1	| Ribosomal protein L22p/L17e family prote	66(11)			
444	AT1G20620.1	CAT3, SEN2, ATCAT3 | catalase 3 | chr1:7	66(3)			
445	AT1G20620.2	CAT3, SEN2, ATCAT3 | catalase 3 | chr1:7	66(3)			
446	AT1G20620.4	CAT3, SEN2, ATCAT3 | catalase 3 | chr1:7	66(3)			
447	AT1G20620.5	CAT3, SEN2, ATCAT3 | catalase 3 | chr1:7	66(4)			
448	AT4G27520.1	ENODL2, AtENODL2 | early nodulin-like pro	66(3)			
449	AT3G01740.1	| Mitochondrial ribosomal protein L37 | ch	64(2)		51(3)	
450	AT5G15200.2	| Ribosomal protein S4 | chr5:4935602-493	64(6)			
451	AT1G06680.2	PSBP-1, OEE2, PSII-P | photosystem II sub	64(3)			
452	ATCG00650.1	RPS18 | ribosomal protein S18 | chrC:6791	64(4)			
453	AT5G61170.1	| Ribosomal protein S19e family protein |	63(5)			
454	AT3G06270.1	| Protein phosphatase 2C family protein |	63(3)			
455	AT2G30870.1	ATGSTF10, ERD13, ATGSTF4, GSTF10 | gluta	61(4)			
456	AT1G52520.1	FRS6 | FAR1-related sequence 6 | chr1:19	60(3)			
457	AT5G25910.1	AtRLP52, RLP52 | receptor like protein 52	60(4)			
458	AT1G54010.1	| GDSL-like Lipase/Acylhydrolase superfamil	59(4)			
459	AT3G02080.1	| Ribosomal protein S19e family protein | c	58(3)			
460	AT3G05900.1	| neurofilament protein-related | chr3:1761	57(3)			
461	AT5G24780.1	VSP1, ATVSP1 | vegetative storage protein	57(3)			
462	AT5G19500.1	| Tryptophan/tyrosine permease | chr5:657	56(3)			
463	AT5G67410.1	| unknown protein; BEST Arabidopsis thaliana p	56(4)			
464	AT2G17060.1	| Disease resistance protein (TIR-NBS-LRR	56(3)			
465	AT1G67120.1	| ATPases;nucleotide binding;ATP binding;nuc	54(6)			
466	AT3G28200.1	| Peroxidase superfamily protein | chr3:10	53(4)			
467	AT4G31570.1	| CONTAINS InterPro DOMAIN/s: Prefoldin (In	52(6)			
468	AT5G15520.1	| Ribosomal protein S19e family protein | c	52(3)			
469	AT3G03590.1	| SWIB/MDM2 domain superfamily protein |	52(5)			
470	AT1G14890.1	| Plant invertase/pectin methylesterase inhi	52(4)			
471	AT2G25720.1	| unknown protein; Has 38 Blast hits to 38 pr	51(5)			
472	AT5G17390.1	| Adenine nucleotide alpha hydrolases-like	51(4)			
473	AT2G35880.1	| TPX2 (targeting protein for Xklp2) protein	51(3)			
474	AT2G42800.1	AtRLP29, RLP29 | receptor like protein 29	51(4)			
475	AT3G09200.2	| Ribosomal protein L10 family protein | ch	50(4)			
476	AT3G09200.1	| Ribosomal protein L10 family protein | ch	50(4)			
477	AT3G16780.1	| Ribosomal protein L19e family protein |	50(3)			
478	AT3G11250.1	| Ribosomal protein L10 family protein | c	50(3)			
479	AT2G40010.1	| Ribosomal protein L10 family protein | ch	50(3)			
480	AT3G14860.2	| NHL domain-containing protein | chr3:49	50(3)			
481	AT3G14860.1	| NHL domain-containing protein | chr3:49	50(3)			

482	AT5G54110.1	ATMAMI, MAMI | membrane-associated mannit	50(3)			
483	AT1G79550.2	PGK | phosphoglycerate kinase | chr1:2992			134(3)	
484	AT1G79550.1	PGK | phosphoglycerate kinase | chr1:2992			134(3)	
485	AT3G03150.1	| unknown protein; FUNCTIONS IN: molecular_			119(4)	
486	AT1G70370.2	PG2 | polygalacturonase 2 | chr1:26513003			62(3)	
487	AT1G70370.1	PG2 | polygalacturonase 2 | chr1:26513003			62(3)	
488	AT1G59760.1	| RNA helicase, ATP-dependent, SK12/DOB1			59(3)	
489	AT5G46260.1	| disease resistance protein (TIR-NBS-LRR c			56(3)	
490	AT1G22690.3	| Gibberellin-regulated family protein | ch		417(8)		
491	AT1G22690.2	| Gibberellin-regulated family protein | ch		417(8)		
492	AT1G22690.1	| Gibberellin-regulated family protein | ch		417(8)		
493	AT3G06700.2	| Ribosomal L29e protein family | chr3:211		325(3)		
494	AT3G06700.3	| Ribosomal L29e protein family | chr3:211		325(3)		
495	AT3G06700.1	| Ribosomal L29e protein family | chr3:211		325(3)		
496	AT1G75750.2	GASA1 | GAST1 protein homolog 1 | chr1:2		322(5)		
497	AT1G75750.1	GASA1 | GAST1 protein homolog 1 | chr1:2		322(5)		
498	AT5G14920.1	| Gibberellin-regulated family protein | ch		307(7)		
499	AT3G26740.1	CCL | CCR-like | chr3:9827868-9828461 F		91(3)		
500	AT3G18070.2	BGLU43 | beta glucosidase 43 | chr3:6187		72(3)		
501	AT3G18070.1	BGLU43 | beta glucosidase 43 | chr3:6187		72(3)		
502	AT2G38530.1	LTP2, LP2, cdf3 | lipid transfer protein 2		60(3)		
503	AT1G16210.1	| unknown protein; CONTAINS InterPro DOMA		56(4)		
